# The TyrA family of aromatic-pathway dehydrogenases in phylogenetic context

**DOI:** 10.1186/1741-7007-3-13

**Published:** 2005-05-12

**Authors:** Jian Song, Carol A Bonner, Murray Wolinsky, Roy A Jensen

**Affiliations:** 1 Los Alamos National Laboratory, Los Alamos, New Mexico, 87545, USA; 2 Emerson Hall, University of Florida, P.O. Box 14425, Gainesville, Florida, 32604-2425, USA

## Abstract

**Background:**

The TyrA protein family includes members that catalyze two dehydrogenase reactions in distinct pathways leading to L-tyrosine and a third reaction that is not part of tyrosine biosynthesis. Family members share a catalytic core region of about 30 kDa, where inhibitors operate competitively by acting as substrate mimics. This protein family typifies many that are challenging for bioinformatic analysis because of relatively modest sequence conservation and small size.

**Results:**

Phylogenetic relationships of TyrA domains were evaluated in the context of combinatorial patterns of specificity for the two substrates, as well as the presence or absence of a variety of fusions. An interactive tool is provided for prediction of substrate specificity. Interactive alignments for a suite of catalytic-core TyrA domains of differing specificity are also provided to facilitate phylogenetic analysis. *tyrA *membership in apparent operons (or supraoperons) was examined, and patterns of conserved synteny in relationship to organismal positions on the 16S rRNA tree were ascertained for members of the domain *Bacteria*. A number of aromatic-pathway genes (*hisH*_*b*_, *aroF*, *aroQ*) have fused with *tyrA*, and it must be more than coincidental that the free-standing counterparts of all of the latter fused genes exhibit a distinct trace of syntenic association.

**Conclusion:**

We propose that the ancestral TyrA dehydrogenase had broad specificity for both the cyclohexadienyl and pyridine nucleotide substrates. Indeed, TyrA proteins of this type persist today, but it is also common to find instances of narrowed substrate specificities, as well as of acquisition via gene fusion of additional catalytic domains or regulatory domains. In some clades a qualitative change associated with either narrowed substrate specificity or gene fusion has produced an evolutionary "jump" in the vertical genealogy of TyrA homologs. The evolutionary history of gene organizations that include *tyrA *can be deduced in genome assemblages of sufficiently close relatives, the most fruitful opportunities currently being in the Proteobacteria. The evolution of TyrA proteins within the broader context of how their regulation evolved and to what extent TyrA co-evolved with other genes as common members of aromatic-pathway regulons is now feasible as an emerging topic of ongoing inquiry.

## Background

**Figure 1:**
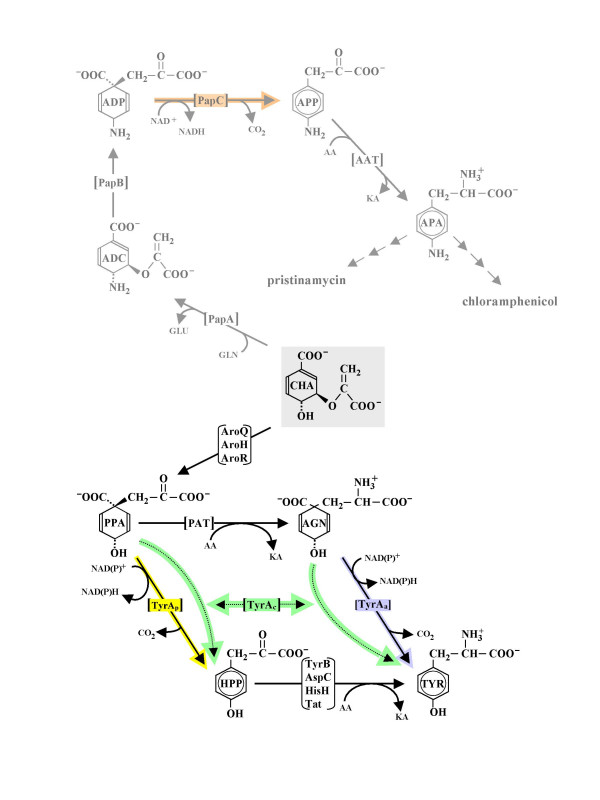
Composite of alternative biochemical routes from chorismate (CHA) to L-tyrosine (TYR) in nature. An antibiotic synthesis branch from CHA is also shown (dimmed). Here the intermediates shown to intervene between chorismate and pristinamycin or chloramphenicol are *p*-aminochorismate (ADC), *p*-aminoprephenate (ADP), *p*-aminophenylpyruvate (APP), and *p*-aminophenylalanine (APA). PPA may be transaminated by prephenate aminotransferase (PAT) to yield *L*-arogenate (AGN). The four TyrA homologs and the reactions they catalyze are colored differently. Arogenate dehydrogenase (TyrA_**a**_) converts AGN to TYR. Alternatively, prephenate dehydrogenase (TyrA_**p**_) converts PPA to 4-hydroxyphenylpyruvate (HPP) which is then transaminated to TYR via an homolog of TyrB, AspC, HisH, or Tat [49]. A broad-specificity cyclohexadienyl dehydrogenase (TyrA_**c**_) is competent to catalyze either the TyrA_**a **_or the TyrA_**p **_reaction. PapC converts the 4-amino analog of PPA to the 4-amino analog of HPP. AroQ, AroH, and AroR are distinct homologs known to exist in nature for performance of the chorismate mutase reaction. Other abbreviations: AA, amino acid donor, KA, keto-acid accepter.

Dehydrogenases dedicated to L-tyrosine (TYR) biosynthesis comprise a family of TyrA homologs that have different specificities for the cyclohexadienyl substrate: ones specific for L-arogenate (AGN), ones specific for prephenate (PPA), and those that are able to use both [1,2]. Figure 1 illustrates the biochemical relationship of these specificities to divergent transformations beginning with chorismate (CHA) utilization and converging on TYR formation. Compounding this complexity, a given TyrA enzyme having any of the aforementioned cyclohexadienyl specificities may be specific for NAD^+ ^or NADP^+^, or may use both. This is consistent with a growing appreciation [3,4] that different substrate specificities are often accommodated across a given protein family that nevertheless maintains a common scaffold of fundamental reaction chemistry. Even within the single category of broad TyrA specificity, there is a continuum ranging from examples where alternative substrates are accepted equally well to other cases where one substrate may be preferred by an order of magnitude or more. Table 1 provides a key to the nomenclature used to identify the various possible substrate-utilization combinations (both cyclohexadienyl and pyridine nucleotide) exhibited by TyrA proteins.

The TyrA family is typical of many protein families in that its members have a relatively small core domain that is not highly conserved. As such, substantial challenges for bioinformatic analysis are posed. Here we have not only carried out a labor-intensive manual analysis, but we have also developed tools intended to facilitate and refine follow-on studies of this protein family in the genome era. The approaches implemented in this study with the TYR segment of aromatic biosynthesis hopefully can serve as a template for forthcoming integrant analyses of other pathway segments of aromatic biosynthesis, and indeed for metabolic subsystems in general.

This manuscript contains three broad sections. First, the biochemical and enzymological complexity of the TyrA protein family is presented in terms of the diversity that exists in nature with respect to substrate specificity and the association of the core domain with other catalytic or regulatory domains. Secondly, the genomic colinear organization of *tyrA *genes with other genes is evaluated, i.e., *tyrA *is considered in its syntenic context. Thirdly, *tyrA *is evaluated in its context of regulation. These three sections are tied together in a framework of evolutionary perspective.

## Results and discussion

### Background of TyrA diversity

**Figure 2:**
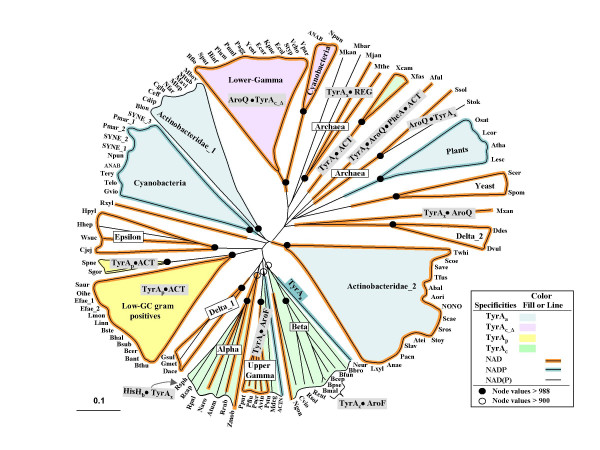
Phylogenetic tree for trimmed core domains of selected members of the TyrA Superfamily. Acronyms used for the various organisms are given in alphabetical order in Table 2. (A more extensive listing that includes organisms not shown in Fig. 2 and which also is hyperlinked to all of the individual GenBank records is given in Table S1. A similar table that also includes compilation of known and predicted substrate specificities is maintained at AroPath [73]. Lineages possessing experimentally established TyrA_**a**_, TyrA_p_, TyrA_c _or TyrA_c_Δ _proteins are indicated by fill colors specified in the legend. Three specificity patterns for the pyridine nucleotide substrate are shown by line colors (see figure box). Although the cyanobacteria are depicted as having NADP^+^-specific TyrA proteins, some of them can also accept NAD, albeit to a lesser degree. All proteins having an aspartate residue homologous to D-32 of the *E. coli *_NAD_TyrA_c_Δ _domain are presumed specific for NAD^+^. Fusion of TyrA domains with other catalytic domains is indicated within grey boxes (AroQ•TyrA, TyrA•AroF, HisH_b_•TyrA, and TyrA•AroQ•PheA•ACT) using the convention of a bullet to represent the interdomain area. The boxes overlap any relevant lineages. TyrA proteins having carboxy-terminal fusions with regulatory domains (TyrA•ACT and TyrA•REG) are also shown. The distance scale bar at the bottom left represents substitutions per site.

Our evolutionary analysis is limited by the amount of information that can be managed in a single study, with the focus fixed upon the domain *Bacteria *(due to the relative density of genome representation for *Bacteria *in the public databases). However, in order to show where future expansion of the analysis might lead, the selection of TyrA proteins in Fig. 2 are from all three domains of life, i.e., *Bacteria*, *Archaea*, and *Eukarya *(lower eukaryotes and higher plants). For practicality of presentation, numerous orphan (i.e., without close relatives) TyrA sequences are not shown, and not all members of a given group are necessarily included. The main purposes of the radial tree shown in Fig. 2 are: (i) to illustrate that TyrA proteins of major phylogenetic groupings are generally congruent with 16S rRNA groupings and (ii) to convey a snapshot visualization of the overall complexity of the TyrA protein family from the vantage point of its varied substrate specificities as well as its multiple fusion partners.

As an illustration of the detailed information that follows, note that the TyrA sequences from the beta Proteobacteria at five o'clock in Fig. 2 form a cohesive cluster (termed a 'congruency group'). In this clade there exists a proposed ancestral background of broad specificity where either AGN or PPA in combination with either NAD^+ ^or NADP^+ ^could be used. This profile of broad substrate use (which can be denoted as _NAD(P)_TyrA_c_; see Table 1) generally persists in the beta Proteobacteria. From this background, narrowed specificities for the AGN/NADP^+ ^couple emerged once in the lineage represented by *Nitrosomonas europaea *(Fig. 2; dark blue line), narrowed specificity for NAD^+ ^emerged once in species of *Neisseria *(orange line), and fusion of *tyrA*_*c *_with *aroF *(which encodes enolpyruvylshikimate-3-P synthase, the sixth enzyme in the common pathway of aromatic biosynthesis; see [5,6] for nomenclature used) occurred recently within the *Burkholderia *lineage. These character-state transformations appear to occur with relative ease, and independent emergence of the same character states can be seen elsewhere in the tree.

### Phylogenetically congruent TyrA groupings

#### Multiple alignments of catalytic-core domains

A phylogenetic tree is only as good as the input alignment. An optimal multiple alignment of TyrA homologs requires a trimmed set of sequences that corresponds to the catalytic-core domain. Alignment of sequences with non-homologous N-terminal fusions (such as with chorismate mutase• (AroQ•), HisH_b_•, or plant transit peptides•; note the convention of using a bullet to indicate the fusion point of one domain with another domain) will make them appear to be more closely related than they actually are because residues in the non-homologous N-terminal regions find matches at random. Likewise, those TyrA sequences with C-terminal fusions (such as with •AroF, •ACT, or •REG) will appear to be anomalously close to one another. Even enzyme proteins that have much greater sequence conservation and amino-acid lengths than TyrA proteins cannot reasonably be expected to yield a protein tree that would be congruent over an extensive phylogenetic range with the overall 16S rRNA tree. However, if genome representation is sufficiently dense within a range of closely related organisms, 16S rRNA congruency with a given protein can be expected within that range of organisms provided that (i) the particular functional role has been retained and (ii) lateral gene transfer has not occurred to obscure the relationship. This expectation follows from the outcome of a detailed analysis of tryptophan-pathway proteins in *Bacteria *[7,8].

#### Congruency within major clades

TyrA sequences from higher-plant and yeast *Eukarya *form cohesive clusters. Genome representation among *Archaea *is still relatively limited. (Fig. 2 does reveal, however, that genes encoding TyrA proteins in *Archaea *have experienced various catalytic- and regulatory-domain fusions at least as frequently as those in *Bacteria*). Eventual expansion of both the tryptophan-pathway and tyrosine-pathway analyses to *Archaea *should be quite interesting.

The great majority of TyrA sequences available are from *Bacteria*, and one can see (by inspection of the major clades supported by high bootstrap values in Fig. 2) a qualitatively apparent congruence of TyrA-tree sub-sections with 16S rRNA expectations of vertical genealogy. Thus, all cyanobacteria possess a _NADP_TyrA_a _type of TyrA enzyme, and this is a very cohesive grouping. A few of the larger cyanobacterial genomes have a co-existing second enzyme of the TyrA_c_Δ _type (discussed in detail later). The low-GC gram-positive bacteria (*Bacillus/Staphylococcus/Enterococcus/Listeria*) exhibit the _NAD_TyrA_p _pattern of specificity and also possess a C-terminal domain (ACT) of allosteric regulation. It is interesting that the TyrA_p_•ACT proteins of the *Streptococcus *lineage (at eight o'clock in Fig. 2) differ from the main low-GC clade in possessing broad specificity for pyridine nucleotides (as indicated with black line color). The most parsimonious evolutionary conclusion would be that in the low-GC gram-positive grouping, acquisition of the ACT domain and narrowed specificity for prephenate preceded narrowed specificity for NAD^+^. Thus, the latter event occurred after divergence of the *Streptococcus *lineage from the remainder of the low-GC clade. Members of the subclass taxon *Actinobacteridae *(mostly actinomycetes) possess AGN-specific TyrA enzymes (light blue fill color in Fig. 2), but they separate into two distinct groups that correlate either with broad specificity for pyridine nucleotides (*Actinobacteridae*_1) or a NAD^+^-specific pattern (*Actinobacteridae*_2). The Proteobacteria are discussed immediately below.

#### Proteobacteria

By far the greatest genomic density available is for Proteobacteria, the group of *Bacteria *that includes purple bacteria and their relatives. The various divisions of Proteobacteria, as currently named, lack hierarchical equivalence. For example, the epsilon and delta divisions branch from much deeper positions on the phylogenetic tree than do the alpha Proteobacteria. As genome representation expands for epsilon and delta Proteobacteria, it is probable that these will subdivide to newly named groupings of approximate hierarchical equivalence with alpha Proteobacteria. The most recently diverged Proteobacteria are the beta and gamma divisions. From the combination of our previous analysis of tryptophan biosynthesis [7,8], TYR biosynthesis (this paper), and other segments of aromatic biosynthesis (unpublished data), we find it useful to separate "upper-gamma" Proteobacteria from "lower-gamma" Proteobacteria (an "enteric lineage" with *Shewanella oneidensis *as approximately the most divergent member). This separation is because the beta Proteobacteria and the upper-gamma Proteobacteria exhibit a smooth continuity of relatively few evolutionary events with respect to aromatic biosynthesis, in striking contrast to extraordinarily dynamic evolutionary events in the lower-gamma Proteobacteria. As a consequence, the lower-gamma Proteobacteria are much more distinct (in terms of aromatic biosynthesis) from the upper-gamma Proteobacteria than the upper-gamma are from the beta Proteobacteria.

Figure 2 shows that alpha, beta and epsilon divisions of Proteobacteria form phylogenetically coherent clusters with respect to their TyrA proteins. Although delta Proteobacteria fall into two well-separated groupings denoted as Delta_1 and Delta_2, this should not be surprising since these groupings diverge at a deep level on the 16S rRNA tree where genome representation is poor. In addition, the *Myxococcus xanthus *TyrA sequence, currently an orphan (three o'clock in Fig. 2), represents a third divergent lineage in delta Proetobacteria. In contrast to delta Proteobacteria, genomic representation for the gamma Proteobacteria is relatively good. Nevertheless their TyrA sequences separate into several well-spaced groupings, albeit for entirely different reasons. In this case, the split seen between two clades of these fairly close relatives (upper-gamma and lower-gamma) is attributed to particularly dynamic evolutionary events compressed into a relatively short time span in the lower-gamma Proteobacteria. (We refer to such a dynamic divergence as an evolutionary jump; see the next section.) Note that the allocation of upper-gamma and lower-gamma Proteobacteria to separate TyrA congruency groups is not the same as being incongruent. It is quite possible that as new genomes come on line, new and intermediate TyrA sequences may result in the merging of the foregoing two congruency groups (currently tyrosine congruency group 1 (TyrCG-1) and tyrosine congruency group 2 (TyrCG-2)).

#### Comparison of tryptophan and tyrosine congruency groups

Although the true extent of lateral gene transfer (LGT) at present must be described as intensely controversial, there is little doubt that any given organism is mosaic with respect to some unknown fraction of its gene repertoire. Our "accounting" system for keeping track of proteins that are faithful to the vertical genealogy is to formulate congruency groupings that are defined by congruence of given protein-tree clusters to a section of the 16S rRNA tree. Ultimately this information will reveal which organisms are "pure" with respect to the vertical inheritance of a given pathway or pathway segment. Our congruency groups are intended to be fluid, in that with the continued availability of new sequences, a previous orphan sequence may very well become the seed for a new congruency group. On the other hand, previously separate congruency groups have the potential to merge. (See Methods for more information.) The present tyrosine congruency groups are listed on the AroPath website [9].

Seven tryptophan congruency groups in *Bacteria *were previously formulated [8] based upon the correspondence of cohesive clusters in trees of Trp-protein concatenates with sections of 16S rRNA trees. The information input for formulation of tryptophan congruency groups is of greater quality than for tyrosine congruency groups because seven-protein concatenates could be used for the former. On the other hand, the broad information input supporting tyrosine congruency groups in this study is more comprehensive because of greater genome availability. Tryptophan congruency group 1 (TrpCG-1) corresponds perfectly with the organisms represented in TyrCG-1, these being the lower-gamma Proteobacteria (enteric lineage). The upper-gamma Proteobacteria (TyrCG-2) and the beta Proteobacteria (tyrosine congruency group 3; TyrCG-3) are represented by different tyrosine congruency groups. In contrast, the membership of tryptophan congruency group 2 (TrpCG-2) includes both the upper-gamma Proteobacteria and the beta Proteobacteria. The latter merging probably reflects the advantage conferred by the greater information content of the concatenated sequences used to define tryptophan congruency groups.

Species of *Xylella *and *Xanthomonas *are usually referred to as gamma Proteobacteria. They probably represent an outlying deeply branching lineage, although trees based on concatenated strings of proteins [10] or 16S rRNA [11] position them with beta Proteobacteria. In any event, Trp-protein concatenate trees placed *Xylella *and *Xanthomonas *within TrpCG-2, which contains both upper-gamma and beta Proteobacteria. In contrast, the TyrA domains from *Xylella *and *Xanthomonas *were well separated (at about two o'clock in Fig. 2) from those of any other organism. This might simply be due to the limited resolving power of a single protein in combination with too few close relatives. (Note that single Trp-protein trees sometimes failed to achieve the congruency-group placements that were resolved by seven-protein Trp concatenates [8]). An additional clue may be relevant. The TyrA proteins from the *Xylella/Xanthomonas *genera possess an ACT domain, which has not been observed in any other proteobacterial TyrA proteins thus far. In view of this, origin by LGT seems to be a distinct possibility, but with the important caveat that no likely genome donors are yet obvious on the criterion of sequence similarity. Perhaps more likely is the following possible explanation that postulates a basis for accelerated divergence. The TyrA domains of *Xanthomonas/Xylella *proteins have an indel structuring (insertions and/or deletions) that places them within the TyrA_c_Δ _specificity subclass (see below). We suggest (see below) that such indel structuring reflects interaction of the core TyrA domain with an extra-domain extension. Thus, selection for amino acid changes accomplishing a new domain-domain interaction could account for accelerated divergence of the *Xanthomonas/Xylella *sequences on the TyrA tree (Fig. 2).

Cohesive tryptophan congruency groups of the alpha Proteobacteria (tryptophan congruency group 3; TrpCG-3) and the cyanobacteria (tryptophan congruency group 4; TrpCG-4) match up well with the corresponding tyrosine congruency groups (tyrosine congruency group 4 (TyrCG-4) and tyrosine congruency group 8 (TyrCG-8), respectively). The TyrA proteins of epsilon Proteobacteria define a cohesive tyrosine congruency group (tyrosine congruency group 5; TyrCG-5), whereas the Trp-protein concatenates of epsilon Proteobacteria did not exhibit a coherent congruency group, due at least in part to LGT [8]. The delta Proteobacteria separate into two distinct tyrosine congruency groups: Delta_1 (tyrosine congruency group 6; TyrCG-6) and Delta_2 (tyrosine congruency group 7; TyrCG-7), as shown in Fig. 2. It is likely that corresponding tryptophan congruency groups exist (work in progress), but at the time of the Xie et al. study [8] only Trp-pathway protein concatenates for *Desulfovibrio vulgaris *(Delta_2) and *Geobacter sulfurreducens *(Delta_1) were available, and they were provisionally listed as "orphans". In the present work TyrA sequences from *Deinococcus radiodurans *and *Thermus thermophilus *are the sole members of tyrosine congruency group 12 (TyrCG-12). At the time of the Trp-pathway work, the genome of *Thermus *was unavailable and the *Deinococcus *concatenate was listed as an orphan. It is expected that the *Deinococcus *and *Thermus *concatenates will now seed a new tryptophan congruency group.

Whereas tryptophan congruency group 5 (TrpCG-5) is defined by cohesive concatenates from actinomycete bacteria, the TyrA proteins from the same organisms separated into two distinct congruency groups. It is intriguing that this partitioning into two congruency groups correlates with narrowed specificity for NAD^+ ^(indicating an evolutionary jump) in one of the groups. The latter group (tyrosine congruency group 11; TyrCG-11) is denoted *Actinobacteridae*_2 in Fig. 2, whereas tyrosine congruency group 10 (TyrCG-10) is displayed as *Actinobacteridae*_1. The opposite scenario whereby a single tyrosine congruency group corresponds to split tryptophan congruency groups applies in the case of low-GC gram-positive bacteria. Whereas TyrA proteins form a single congruency group in these organisms (tyrosine congruency group 9; TyrCG-9), a small cluster of Trp-pathway concatenates from *Bacillus subtilis*, *B. stearothermophilus*, and *B. halodurans *(tryptophan congruency group 6; TrpCG-6) separate distinctly from the remaining organisms (tryptophan congruency group 7; TrpCG-7). The latter evolutionary jump reflects a dynamic scenario of tryptophan-pathway evolutionary events that include loss of one gene from the *trp *operon, insertion of the *trp *operon into a 6-gene *aro *operon to produce a supraoperon, and acquisition of the TRAP (tryptophan-activated RNA-binding protein) mechanism of regulation by an RNA-binding protein [7].

Tyrosine congruency groups and tryptophan congruency groups are maintained and updated at the AroPath website [12].

### Distribution in nature of TyrA specificity subclasses for the cyclohexadienyl substrate

Four qualitative classes of specificity for the cyclohexadienyl substrate populate the TyrA superfamily of homologs (Fig. 1). These include PPA-specific (TyrA_p_), AGN-specific (TyrA_a_), the broad-specificity cyclohexadienyl (TyrA_c_) dehydrogenases and a fourth class represented by an enzyme of antibiotic biosynthesis (PapC) that converts 4-amino-4-deoxy-prephenate to 4-amino-phenylpyruvate [13]. Representatives of each specificity class have been studied at molecular and genetic levels. TyrA family members sharing a given substrate specificity do not necessarily cluster tightly together, and assignment of substrate specificity to experimentally uncharacterized TyrA homologs is uncertain unless they exhibit very high amino acid identities with experimentally characterized TyrA proteins. In some cases we do not accept older literature reports without more recent verification. For example, the yeast *Saccharomyces cerevisiae *TyrA_**x **_was characterized as a TyrA_**p **_protein [14] long before it was recognized [15] that PPA preparations were often contaminated with AGN (an unknown compound at that time).

**Figure 3:**
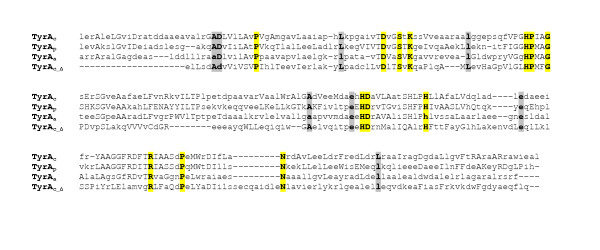
Multiple alignment of the HMM consensus sequences obtained for different substrate-specificity groupings within cyclohexadienyl-substrate core segments (see Table 3). Invariant anchor residues are highlighted in yellow, conserved residues in grey. These consensus sequences will change continuously as corrections and refinements are made. The version shown was current as of April, 2005.

Our collection of curated TyrA sequences at AroPath (see Table 3) contains trimmed sequences that comprise catalytic-core domains. This collection was divided into two groups based on whether the sequences contained the relatively short N-terminal pyridine-nucleotide discriminator segment or the longer C-terminal cyclohexadienyl-substrate core segment. The sequences in the latter group were assembled into subgroups representing established substrate specificities (TyrA_a_, TyrA_p _and TyrA_c_) and were aligned separately to obtain overall consensus sequences for cyclohexadienyl-substrate core segments. The TyrA_c _group members from the lower-gamma assemblage of Proteobacteria (as well as from a few other lineages) were so distinctive that a fourth group (TyrA_c_Δ_) was defined. This latter group is, in fact, the most divergent of the four. Figure 3 shows a comparison of the four consensus sequences, with invariant anchor residues shaded yellow and residues conserved across all groups shaded in gray. Residues within each group that are >50% conserved are shown in capital letters. In pairwise BLAST (Basic Local Alignment Tool) [16]comparisons, TyrA_a _and TyrA_c _consensus sequences are most similar (47% identity), followed by the TyrA_c_/TyrA_p _pair (40% identity), with TyrA_a _and TyrA_p _exhibiting 34% identity. TyrA_c_Δ _is quite distinct from the other three groupings, exhibiting only 27% identity with TyrA_c_, 23% identity with TyrA_c_, and 18% identity with TyrA_p_.

#### Cyclohexadienyl dehydrogenases

Many TyrA proteins (at least in the domain *Bacteria*) are of the TyrA_c _subclass. The cyclohexadienyl dehydrogenases commonly accept PPA or AGN about equally well, but various degrees of preference for one of the alternative substrates are also observed. Detailed molecular and genetic studies of TyrA_c _proteins from *Pseudomonas aeruginosa*, [17], *P. stutzeri *[1], and *Zymomonas mobilis *[18] have been carried out. The distinct variety of TyrA_c _mentioned above, which has been denoted TyrA_c_Δ _exhibits a number of indels (mostly deletions) within the catalytic-core region when its consensus sequence is aligned with those of the other TyrA classes (Fig. 3). It is intriguing that the indel structuring of TyrA_c_Δ _correlates with the presence of an extra-core extension. This extension is often AroQ, but not always. For example, in the genera *Nostoc *and *Anabaena *it appears to be a degraded, catalytically inactive AroQ, whereas in *Xanthomonas *or *Xylella *it is an ACT domain. Since the one large clade of TyrA_c_Δ _proteins that has so far been studied prefers PPA over AGN by well over an order of magnitude, an evolutionary relationship of indel insertions to the narrowing of substrate preference for PPA might exist. If so, however, this cannot be the only molecular change to accomplish favored utilization of PPA over AGN since a number of TyrA_c _proteins, (e.g., TyrA_c _from *Neisseria gonorrhoeae*), also exhibits an overwhelming preference for PPA, even though this class lacks the indel structuring.

#### Arogenate dehydrogenases

The TyrA_**a **_class of specificity is currently represented by higher plants and at least three widely spaced bacterial lineages: cyanobacteria, actinomycetes and *Nitrosomonas europaea*. This discontinuity of phylogenetic spacing is consistent with a fundamental evolutionary scenario [19] whereby the ancestral dehydrogenase was a broad-specificity TyrA_**c **_that evolved narrowed substrate specificity (to yield either TyrA_**p **_or TyrA_**a**_) independently on multiple occasions in modern lineages. The ubiquitous presence of TyrA_a _in cyanobacteria has been heavily documented [20]. *Nitrosomonas europaea *currently (as of March, 2005) has no sufficiently close genome relatives that have been sequenced. The first BLAST hit returned from a _NADP_TyrA_**a **_query from *N. europaea *(March,2005) is the protein from *Ralstonia solanacearum *(48% identity), which is known to possess broad specificity for both of its substrates (i.e., _NAD(P)_TyrA_**c**_) [21,22].

**Figure 4:**
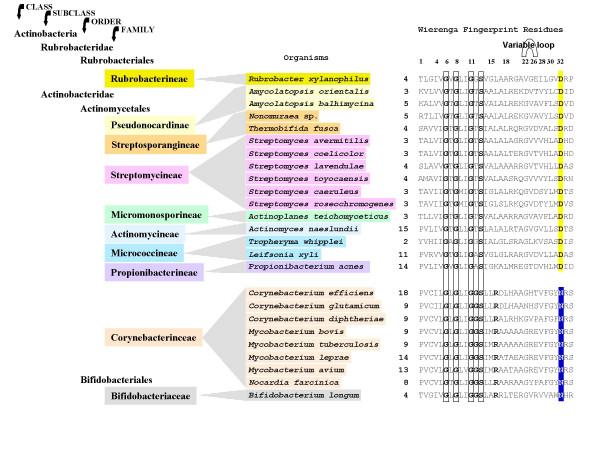
Alignment of the N-terminal glycine-rich P-loop of TyrA•ACT proteins from the Class *Actinobacteria*. These are specific for *L*-arogenate as substrate, but fall into two groups with respect to the pyridine nucleotide co-substrate. The top NAD^+^-specific group possesses an aspartate (D) at position 32 (*E. coli *numbering), whereas the bottom NAD^+^/NADP^+ ^group possesses an asparagine at the homologous position. Residue numbers are shown at the left. The species in the middle are color coded to match the hierarchical taxon positions obtained from NCBI. The variable loop of the Wierenga fingerprint [26], which in *E. coli *contains five residues (22–26), contains the minimal two residues in all of the *Actinobacteria *shown. The organisms on the right are color coded according to the taxonomic position indicated on the left (NCBI). The *Rubrobacter xylanophilus *TyrA_a _sequence is an orphan in the tree displayed in Fig. 2, as consistent with its outlying position in the taxonomy scheme.

The TyrA sequences of *Actinobacteria *separate into two distinct groupings on the protein tree (Fig. 2). Coryneform bacteria in one sub-cluster have been rigorously characterized as the _NAD(P)_TyrA_a _substrate specificity type. On the other hand, a variety of *Streptomyces *species have been shown [23,24] to possess _NAD_TyrA_a_, and TyrA proteins of these organisms populate the second *Actinobacteria *sub-cluster of Fig. 2. Figure 4 shows sequence alignments of the N-terminal pyridine-nucleotide discriminator regions of currently available actinomycetes. The conserved 'D' residue (highlighted in yellow) in the upper group is a reliable indicator of NAD^+ ^specificity, in part because NADP^+ ^is repelled by the negative charge at this position. The asparagine residue (highlighted in blue) in the corresponding position in members of the lower group indicates NAD(P)^+ ^specificity as discussed by Bonner et al. [25]. *Rubrobacter xylanophilus *is the most distant representative of the *Actinobacteria*, being the sole member of the subclass taxon *Rubrobacteridae*, and its protein (denoted Rxyl) appears as an orphan in Fig. 2.

A similar relationship of phylogenetic separation associated with narrowed specificity for pyridine-nucleotide substrate exists for the low-GC gram-positive bacteria (eight o'clock in Fig. 2). Here the major clade is NAD^+^-specific, whereas species of *Streptococcus *have retained the ancestral breadth of specificity for NAD^+^/NADP^+^. Alignments of the pyridine-nucleotide discriminator regions of these latter two groups match up extremely well with the upper alignment of Fig. 4 where residue 32 of the Wierenga fingerprint [26] is 'D' and with the lower alignment where residue 32 is 'N' (data not shown).

Recently, a plant *tyrA*_**a **_from *Arabidopsis thaliana *has been reported to consist of two near-identical domains that are fused [27]. The gene encoding this 68-kDa protein co-exists in the genome with a single-domain paralog [28] that encodes a predicted 37-kDa protein, somewhat larger than the catalytic-core domain of TyrA_**a **_from *Synechocystis*. TyrA_**a **_(known to be located in higher-plant chloroplasts [2]) may have originated from cyanobacteria via endosymbiosis. If so, however, the plant TyrA_a _sequences have diverged sufficiently that they no longer share a specific phylogenetic grouping with the cyanobacterial TyrA sequences. This is in marked contrast with the phylogenetic coherence of the tryptophan synthase subunit proteins (TrpEa and TrpEb_1) from cyanobacteria and higher plants [29].

#### Prephenate dehydrogenases

TyrA_p _is conspicuously represented by a large clade of low-GC gram-positive organisms, of which *Bacillus subtilis *TyrA_p _is the best studied [30]. Thus far, all TyrA_p _proteins are fused to a C-terminal ACT domain, and therefore no "minimal" TyrA_p _proteins that consist only of a catalytic core are available as yet. At the level of physiological function, it should be added that those cyclohexadienyl dehydrogenases that exhibit a very substantial preference for prephenate are for all practical purposes prephenate dehydrogenases, even though they carry a formal designation of TyrA_c _or TyrA_c_Δ_. These include most, if not all, of the AroQ•TyrA_c_Δ _enzymes of the enteric lineage (lower-gamma in Fig. 2). The TyrA_c _protein from *Neisseria gonorrhoeae *(and by inference, the closely related *N. meningitides*) is also a well-studied example of overwhelming preference for prephenate [21].

#### PapC dehydrogenases

PapC participates in the formation of *p*-aminophenylalanine as a step in the synthesis of at least two antibiotics (see Fig. 1). It is so far represented by only a few sequences. The PapC specificity is strongly indicated by absence of the otherwise invariant residue H197 (*E. coli *numbering) that is associated with recognition of a 4-hydroxy moiety in the cyclohexadienyl substrates of the aforementioned dehydrogenases. This moiety, of course, differs in being a 4-amino substituent in the substrate used by the PapC dehydrogenase (Fig. 1). See Bonner et al. [25] for a more detailed overview.

### The "redundant" *trp/aro *supraoperon of *Nostoc/Anabaena*

All cyanobacteria possess a highly conserved *tyrA*_*a *_gene, as well as a complete suite of tryptophan-pathway genes that are dispersed (unlinked) in the genome. The large-genome cyanobacterial lineage consisting of the *Nostoc *and *Anabaena *genera possess in addition a unique and seemingly redundant *trp*/*aro *supraoperon consisting of most of the aforementioned genes [31]. These include a second *tyrA *gene (curated as *tyrA*_*c_Δ*_), six *trp*-pathway genes (all except *trpC*), and genes encoding the first two common-pathway steps of aromatic amino acid biosynthesis. All of these supraoperonic genes appear to be redundant in that they are represented by homologs (paralogs or xenologs) elsewhere in the *Nostoc *and *Anabaena *genomes at scattered loci. The closest BLAST hits for the *Nostoc/Anabaena *TyrA_c_Δ _proteins are not the co-existing TyrA_a _homologs present in their own genomes (and universally present in cyanobacteria). Rather the closest BLAST hits are to the TyrA_c_Δ _domains of the AroQ•TyrA_c_Δ _fusions in the enteric lineage. Since the enteric proteins are NAD^+^-specific and strongly prefer prephenate, it is likely that the "extra" cyanobacterial proteins are also _NAD_TyrA_c_Δ _proteins. Indeed, this would be consistent with enzymological evidence provided in the literature for both *Nostoc *and *Anabaena *[20].

Concerning the evolutionary origin of the redundant block of linked genes found in the *Nostoc *and *Anabaena *genomes, at least two possibilities await further illumination. (i) These genes might have been acquired by a common ancestor of *Nostoc *and *Anabaena *via lateral gene transfer. This is consistent with the observation that biosynthetic-pathway operons are generally absent in the cyanobacteria, and all of the linked genes could have been recruited in a single event. However, at present no candidate donor genomes are known that possess this supraoperon combination of genes. If the TyrA_c_Δ _proteins of *Nostoc*/*Anabaena *and the enteric lineage are possibly related by LGT, it is of interest that the N-terminal extension of TyrA_c_Δ _from *Nostoc*/*Anabaena *resembles a degraded AroQ domain of AroQ•TyrA_c_Δ _from enterics. In both cases the N-terminal residues may compensate for indel deletions within the catalytic core region of TyrA_c_Δ_. Subsequently, AroQ function may have evolved in one lineage (or have been lost in the other). This possibility of domain-domain interaction is consistent with the established interdependence of the AroQ• and •TyrA_c_Δ _domains from *E. coli *[32]. Alternatively, *tyrA*_*a *_and *tyrA*_*c_Δ *_(and the duplicated *trp *and *aro *genes present in the supraoperon) might be ancient paralogs within the cyanobacterial lineage. If so, at a time following divergence of heterocystous cyanobacteria from the unicellular cyanobacteria, the latter may have lost the clustered block of aromatic-pathway genes in a single event of reductive evolution. The supraoperonic genes might be related to a specialized function associated with "developmental" physiological processes that typify the filamentous, heterocyst-forming cyanobacteria. This might be reminiscent of the nature of the phenazine-pigment operon of *Pseudomonas aeruginosa*. Here unique phenazine-pathway genes are combined with a redundant gene of common-pathway aromatic biosynthesis and two redundant (and fused) genes of tryptophan biosynthesis. This accomplishes the linkage of specific phenazine biosynthesis with a supply of 2-amino-2-deoxy-isochorismate, the branchpoint of divergence toward phenazine and tryptophan [33,34]. This complexity in which multiple paralogs are differentially deployed is consistent with the large genome sizes of *Anabaena *(7.2 MB) and *Nostoc *(9.2 MB), compared with the much smaller unicellular genomes of *Prochlorococcus marinus *(1.7 MB), *Synechococcus *sp. WH8102 (2.4 MB), and *Synechocystis *sp. PCC6803 (3.6 MB).

### Profile hidden Markov models (HMMs) to distinguish specificity subfamilies for cyclohexadienyl substrate

The limited information thus far available about specific molecular roles of particular TyrA amino acid residues has been summarized recently [25]. The catalytic-core domains of known TyrA_a_, TyrA_p_, TyrA_c_, and TyrA_c_Δ _proteins were selected from our files of TyrA catalytic-core domains [35], and a new subset of sequences was prepared that lacked the pyridine nucleotide discriminator segment, a glycine-rich βαβ region at the N terminus. Although the glycine-rich βαβ region is not the only segment that contacts pyridine nucleotide substrate, it is the sole region that discriminates between NAD^+ ^and NADP^+^. The resulting trimmed sequence is defined as the "cyclohexadienyl-substrate core segment". No distinctive motifs were found that, in isolation, would be a clear predictive indicator of specificity for cyclohexadienyl substrate. Similar substrate specificity profiles probably can be dictated by alternative patterns of interplay between different residue combinations.

Because of the rapid accumulation of incorrectly annotated TyrA entries in GenBank and other databases, partly due to the complications of misnaming that are associated with gene fusions and partly to a failure to assimilate published substrate specificities, the use of BLAST does not return reliable annotations with respect to substrate specificity. Even the HMMs used in Pfam [36] and Interpro [37] were not helpful in this case because the HMM deployed in those databases was broadly but incorrectly defined as 'prephenate dehydrogenase (NADP^+^) activity' for all TyrA dehydrogenases (accession number PF02153 in Pfam and entry IPR003099 in Interpro). However, Profile HMM is known to be well suited for modeling a particular sequence family of interest and for finding additional remote homologs [38]. It is reputed to outperform methods that rely only upon pair-wise alignment of homologous residues in predicting protein function [39]. Therefore, profile HMMs were constructed using our multiple sequence alignments of each curated TyrA specificity subfamily, using the HMMER package [38].

The profile HMMs obtained are only tentatively reliable for prediction of substrate specificity. To facilitate ongoing and future functional annotations, we have made our profile HMMs available as a working resource for "specificity prediction" at AroPath [40]. Users can match query sequences against the four profile HMMs to predict the subfamily to which a query sequence belongs. It is anticipated that future experimental data relevant to substrate specificity will facilitate refinement of the prediction program. For example, at present the program predicts that the TyrA sequences from organisms such as *Helicobacter pylori *and *Saccharomyces cerevisiae *belong to the TyrA_a _grouping, and it will be interesting to see whether this holds up to experimental confirmation. It is additionally fascinating that (i) the dehydrogenase from *Archaeoglobus fulgidus *is predicted to belong to the indel-containing TyrA_c_Δ _grouping and (ii) that it possesses a possible cooperatively interacting extra-core domain extension (an AroQ fusion), just as occurs for the large clade of enteric bacteria. If this is relevant, it is even more fascinating that the *Archaeoglobus aroQ *is fused at the C-terminal side of *tyrA*_c_Δ_, rather than at the N terminus as is the case with enteric bacteria.

Users at AroPath [41] can enter query sequences into interactive multiple sequence alignments with any of the four sets of "cyclohexadienyl-substrate core segments" sequences that were used to train the profile HMMs. An ongoing effort is in process to extend the predictor capability to include the pyridine nucleotide substrate as well. One can also align query sequences of interest with either an assemblage of the complete set of curator-approved TyrA catalytic-core TyrA sequences or with any desired subset of seed sequences.

### The catalytic-core domain of TyrA proteins

The simplest set of fully functional TyrA proteins consists only of the catalytic-core domain (about 180 amino acids) [1] and includes the well-characterized TyrA_**c **_enzymes from *Neisseria gonorrhoeae *[21] and *Zymomonas mobilis *[18], as well as TyrA_**a **_from a cyanobacterium [25]. In addition the catalytic-core domain from *Pseudomonas stutzeri *has been engineered for study from a *tyrA*_*c*_•*aroF *fusion [1]. These model core proteins are roughly as divergent from one another on the TyrA protein tree as are the organisms that contain them (Fig. 2). In view of the possibility raised in this paper about inter-domain interactions, the single-domain TyrA proteins are undoubtedly the simplest sources for study of the fundamental properties of the catalytic-core domain.

Xie et al. [1] suggested that in the set of catalytic-core TyrA proteins, inhibitors bind at the catalytic site and exhibit classical competitive inhibition with respect to the particular cyclohexadienyl substrates that can be accepted by a given organism. This model predicts that the specificity for the sidechains of substrates used would parallel the specificity for inhibitor sidechains. The information summarized in Table 4 supports this expectation. Thus, the TyrA_**c **_proteins of *P. stutzeri *and *P. aeruginosa *will accept either a pyruvyl (as with PPA) or an alanyl (as with AGN) sidechain in the alternative substrates used, and this is paralleled by recognition of either a pyruvyl (4-hydroxyphenylpyruvate) or an alanyl (TYR) sidechain in the competent inhibitor structures. In another case, the *N. gonorrhoeae *TyrA_c _exhibits an overwhelming substrate preference for PPA, and consistent with the foregoing, is subject to inhibition by 4-hydroxyphenylpyruvate but not by TYR. A variety of analog inhibitor structures were used by Xie et al. [1] to show that the minimal structure for binding at the substrate-binding site of *P. stutzeri *TyrA_**c **_is a six-membered ring with a 4-hydroxy substituent.

In contrast to the TyrA_**c **_proteins just described, the *Z. mobilis *TyrA_**c **_is totally insensitive to inhibition by either 4-hydroxyphenylpyruvate or TYR. Since both of these compounds lack a 1-carboxy moiety, it is reasonable to assume that the 1-carboxy substituent present in the two substrates accepted may be required for binding at the catalytic center. Thus, although TyrA_c _from *Z. mobilis *will accept the same two substrates as does the TyrA_**c **_from *P. stutzeri*, the greatly different inhibition results suggest that *Z. mobilis *obeys more stringent rules for binding at the catalytic site (i.e., a ring carboxylate must be present).

*Synechocystis *sp. and *Arabidopsis thaliana *TyrA_a _proteins accept as a substrate only AGN, which has an alanyl sidechain. The ring-carboxylate moiety is evidently not absolutely required for binding since these TyrA_a _proteins can recognize TYR (alanyl sidechain) as a competitive inhibitor. In contrast, since *N. europaea *TyrA_a _is not inhibited by TYR, it resembles the *Z. mobilis *TyrA_c _in the putative requirement for a 1-carboxy substituent to secure successful binding at the catalytic site.

In summary, some TyrA proteins probably exercise greater discrimination in their requirement for a 1-carboxy moiety for binding at the catalytic site, and these are insensitive to competitive inhibition by the aromatic reaction products (which lack the 1-carboxy substituent). Other TyrA proteins that require the 1-carboxy moiety for the fundamental catalytic process, but presumably do not require it for binding, will recognize product inhibitors that have the same sidechain as any substrate recognized.

### Specificity for the pyridine nucleotide co-substrate within the TyrA superfamily

NAD^+ ^differs from NADP^+ ^only in that NADP^+ ^has a phosphate group esterified at the 2'-position of adenosine ribose. Therefore, the ability of a dehydrogenase to discriminate between those two lies in the particular enzyme region that contacts the ribose moiety. The glycine-rich region known to constitute the ADP-binding βαβ fold is well known to be this point of contact [26]. This Rossmann β α β fold is inevitably positioned at the extreme N terminus of TyrA proteins, and the typical GXGXXG motif is almost always observed, as illustrated in Fig. 4. This region is helpful for assessment of probable specificities for pyridine nucleotide. One can be fairly sure that TyrA proteins possessing D-32 (*E. coli *numbering, reference [26]) are NAD^+^-specific. A negatively charged residue (D or E) at position 32 is critical for hydrogen binding to the diol group of the ribose near the adenine moiety in NAD^+^-specific enzymes. NADP^+^-specific dehydrogenases cannot tolerate a negatively charged residue at position 32. TyrA proteins that possess an asparagine residue in the corresponding position appear to be broadly specific for both NAD^+ ^and NADP^+ ^as discussed above. No clearcut motif has been identified for NADP^+^-specific TyrA proteins, although at least one positively charged residue is expected in the region just beyond residue 32. By elimination, those sequences lacking D-32 or N-32 are strong candidates for NADP^+ ^specificity. As with the cyclohexadienyl co-substrate, narrowed specificity for NAD^+ ^(or NADP^+^) also seems to have occurred independently on many occasions (some examples given earlier).

The absolute specificity of TyrA_p _proteins for PPA tends to be accompanied by absolute specificity for NAD^+^, as illustrated by the large *Bacillus/Staphylococcus/Listeria/Enterococcus *clade at eight o'clock in Fig. 2. However, it is interesting that species of *Streptococcus *have retained the presumed ancestral breadth of specificity for the pyridine nucleotide substrate. The opposite relationship, whereby absolute specificity for AGN tends to be accompanied by absolute specificity for NADP^+^, is also observed. Here three of the four TyrA_a _lineages described earlier exhibit this pattern. Exceptions, though, are the aforementioned TyrA_a _proteins of *Actinobacteridae_*1 which accept either NAD^+ ^or NADP^+^, as well as the TyrA_a _proteins of the sister *Actinobacteridae*_2 which are specialized for NAD^+ ^[42,43].

The TyrA_c _proteins of most complete-genome organisms thus far have happened to be NAD^+^-specific, and this has been the property of the most rigorously characterized ones (from *Z. mobilis*, *P. stutzeri*, and *P. aeruginosa*). However, it is clear from extensive enzymological surveys [22] that TyrA_c _proteins having broad specificity for NAD^+^/NADP^+ ^are common, examples including species of *Ralstonia *and *Burkholderia*. The spectrum of variation that can exist, even within a clade of organisms that are of fairly close relationship, is illustrated by one striking example. In the pseudomonad clade marked by a common *tyrA•aroF *fusion, the *Acinetobacter *sp. TyrA_c _is NADP^+^-specific [44], whereas the sister subclade *Pseudomonas*/*Azotobacter *exhibits NAD^+ ^specificity (Fig. 2). Here the entire clade marked by a common ancestral fusion shares approximately the same profile of cyclohexadienyl substrate preference, but cofactor specificity has been narrowed in opposite directions.

We had previously suggested that there might be a general structural relationship of substrate pairing that tends to favor interaction between PPA and NAD^+^, on the one hand, and, on the other hand, between the greater positive charge of AGN and the greater negative charge of NADP^+^. These relationships may indeed be favored, but it increasingly appears that any combination can occur.

### Beyond the catalytic core: allosteric domains

Various lineages have acquired an amino acid binding domain known as the ACT domain (pfam01842), which is known to bind a variety of amino acids, thus functioning as an allosteric domain for many proteins including phosphoglycerate dehydrogenase, aspartokinase, acetolactate synthase, phenylalanine hydroxylase, prephenate dehydratase and formyltetrahydrofolate deformylase. Recruitment of this domain by fusion with *tyrA*_**p **_appears to have occurred in a common ancestor of the large *Bacillus*/*Staphylococcus*/*Listeria*/*Enterococcus*/*Streptococcus *assemblage (Fig. 2). It is interesting that *B. subtilis *also possesses a gene encoding a free-standing ACT domain in its genome (incorrectly annotated as *pheB*). An additional fusion of genes encoding an ACT domain and *tyrA *(that arose independently, judging from the widely spaced tree positions) occurred in the common ancestor of *Xanthomonas *and *Xylella*. *Actinobacteria *usually possess a C-terminal extension that probably functions as an allosteric domain. The extension possessed by the *Actinobacteridae*_2 assemblage, which includes *Streptomyces coelicolor *and its relatives, appears to be an ACT domain. On the other hand, it is not all all clear that the C-terminal extension of the *Actinobacteridae*_2 assemblage is an ACT domain. This difference, in addition to the differing specificities for pyridine nucleotide substrate, may have contributed to the overall TyrA_a _divergence observed between the two *Actinobacteridae *groups. There is no correlation between presence of the ACT domain and specificity for cyclohexadienyl substrate since TyrA_**p **_from the *Bacillus *clade is PPA-specific, *Xanthomonas*/*Xylella *TyrA_c _is broadly specific, and *Streptomyces *TyrA_a _is AGN-specific.

*B. subtilis*, which belongs to the large clade having an ACT domain as a carboxy extension, has been extensively characterized [30]. 4-Hydroxyphenylpyruvate is an effective competitive inhibitor, as would be consistent with our proposed effects at the catalytic core for a PPA-specific enzyme. However, TYR, phenylalanine (PHE) and tryptophan were also inhibitors. The violation of the rule that the latter three amino acid inhibitors would not be expected to bind the catalytic core region (because they have alanyl sidechains even though the substrate-binding site only recognizes the pyruvyl sidechain of prephenate) and the finding that some of these were not competitive inhibitors can now be accounted for by the presence of the allosteric ACT domain. A carboxy extension shared by a number of *Archaea *(denoted 'REG' in Fig. 2) is presumably a regulatory domain as well. This is consistent with the recent result of Porat et al. [45] that not only 4-hydroxyphenylpyruvate, but also TYR, inhibited prephenate dehydrogenase activity of *Methanococcus maripaludis*.

### The tyrA gene is a popular fusion partner

#### Fusion with *aroQ*

*tyrA *may be fused with a number of other catalytic domains, each of them relevant to aromatic biosynthesis (Fig. 2). *aroQ *(encoding chorismate mutase) is frequently fused with a number of other aromatic-pathway genes [46]. The lower-gamma Proteobacteria (enteric lineage) located at twelve o'clock in Fig. 2 possess an *aroQ•tyrA*_*c_Δ *_fusion. The fusion physically links chorismate mutase (which forms PPA) with TyrA_c_Δ _(which utilizes PPA). The two protein domains of AroQ•TyrA_c_Δ _may have co-evolved to produce cooperative protein-protein interactions since physical separation of the domains evoked relatively low activities of both activities in *E. coli *[32]. Substantial comparative work shows that the *aroQ•tyrA*_*c_Δ *_fusion has been stably maintained throughout the entire enteric lineage [47]. Exceptions in some genomes lacking this fusion altogether can be attributed to reductive evolutionary loss in pathogens (e.g., *Haemophilus ducreyi*) or endosymbionts (e.g., *Buchnera aphidicola*). An independent *aroQ•tyrA *fusion was generated in the common ancestor of *Sulfolobus solfataricus *and *S. tokodaii *(Fig. 2). Since the TyrA domain of *Sulfolobus *species lacks the indel structure of the TyrA_c_Δ _class, it would be interesting to see whether physical separation of the two domains would yield evidence of independent function, in contrast to the results mentioned just above for *E. coli*.

#### Fusion with *aroF*

Secondly, *tyrA*_c _has been fused with *aroF *on at least two separate occasions in *Bacteria*. (The *aroF *gene encodes enolpyruvylshikimate-3-P synthase, the sixth enzyme in the common pathway of aromatic biosynthesis; see [5,6] for nomenclature used.) One clade includes members of the upper-gamma Proteobacteria: *P. aeruginosa*, *P. syringae*, *P. putida*, *P. stutzeri*, *P. fluorescens *and *Azotobacter vinelandii*. It is interesting that *P. syringae *has experienced a deletion of about 200 residues at the N-terminal region of the AroF domain. This has been coupled with the acquisition of a stand-alone *aroF *gene that is absent in other members of the clade. Interestingly, the latter AroF shows high identity only with AroF from *Agrobacterium tumefaciens*, an alpha proteobacterium. The *A. tumefaciens aroF*, in turn, is unique compared to its α-subdivision relatives, both in having divergent sequence and in being unlinked to *cmk *and *rpsA*. Thus, it seems likely that the incongruence of AroF belonging to both *P. syringae *and *A. tumefaciens *reflects acquisition via LGT from some as yet unknown source. The disruption of the fused *aroF *domain in *P. syringae *is an unusual instance where the catalytic function of one fusion domain has become discarded while the function of the second domain has been retained. It is interesting to consider the possibility that the truncated remnant of the *aroF *fusion domain might be exploitable for use as an innovative source of a new regulatory domain. An additional fusion of *tyrA *with *aroF *has occurred independently within the beta Proteobacteria in the common ancestor of *Burkholderia pseudomallei *and *B. mallei*. This has been very recent since the closely related *B. fungorum *and *B. cepacia *organisms lack the fusion.

It has been suggested that presence of a given fusion may be useful for sorting out clades that diverged from a common ancestor, independent of other methods [48]. Different fusions offer the power of discriminating clades at various hierarchical levels, i.e., nested clades discriminated by nested gene fusions. The *tyrA•aroF *fusion occurred in the common ancestor of the clade that includes the upper-gamma Proteobacteria shown in Fig. 2. One can reasonably assume that relatively close upper-gamma organisms lacking the *tyrA•aroF *fusion diverged from the common ancestor of the fusion clade prior to the fusion event. Such would appear to be the case, for example, with *Acidithiobacillus ferrooxidans*, an outlying member of the upper-gamma Proteobacteria that lacks the fusion. It is reasonable to conclude that the fusion event must have pre-dated the differential specialization for the pyridine nucleotide cosubstrate that distinguishes *Acinetobacter *sp. (NADP^+^-specific) from the large grouping of pseudomonads that are NAD^+^-specific.

#### Fusion with *hisH*_b_

Thirdly, a single organism, *Rhodobacter sphaeroides*, possesses a *hisH*_b_•*tyrA *fusion that must have occurred very recently. *hisH*_b _encodes an aromatic aminotransferase that is closely related to (or sometimes even synonymous with) imidazole acetol phosphate aminotransferase [49]. The *hisH*_*b*_/*tyrA*/*aroF *linkage group is part of a supraoperon in some gram-negative bacteria in which a relatively conserved, yet frequently shuffled gene order is observed [5,6]. Hence, it is reasonable to assume that at the time just prior to fusion, *hisH*_b_, *tyrA *and *aroF *were adjacent. Note that among the fusions currently known, *hisH*_b _and *aroF *are fused to the N-terminal and C-terminal ends of *tyrA*, respectively. It would be interesting to know the substrate specificity of the *R. sphaeroides *TyrA domain. If it is AGN-specific the significance of *hisH*_b _presumably would be to transaminate PPA to form AGN, the substrate used by TyrA_a _(see Fig. 1). On the other hand, if the dehydrogenase is PPA-specific, the significance of the HisH_b _domain would be to transaminate the product of the TyrA_p _reaction. If the enzyme is a TyrA_c _enzyme (as is probable), then HisH_b _likely is competent to catalyze either of the foregoing reactions.

#### Fusion with ACT

The widespread ACT regulatory domain appears to have been acquired by independent fusions at least three separate times judging from the widely separated lineages that possess a TyrA•ACT fusion (Fig. 2). Xie et al. [5] initially noted homologous domains positioned at the N terminus of mammalian phenylalanine hydroxylase and at the C terminus of most microbial prephenate dehydratases. This domain is responsible for phenylalanine-mediated activation and phenylalanine-mediated inhibition of the hydroxylase and dehydratase enzymes, respectively. This domain was later named the ACT domain [50] and shown to be a widely distributed domain family that shares a conserved overall fold. Members of the ACT-domain family possess a wide variety of different ligand-binding capabilities. For example, the ACT domain of 3-phosphoglycerate dehydrogenase binds *L*-serine as a allosteric inhibitor.

#### Fusion with REG

Another putative regulatory domain fused to tyrA (denoted *tyrA•REG*) is thus far restricted to some of the *Archaea*. This domain is a predicted regulatory domain, as described in COG4937.

#### A novel 4-domain fusion

*Archaeoglobus fulgidus *exhibits a striking four-domain fusion consisting of three catalytic domains and a regulatory ACT domain (TyrA•AroQ•PheA•ACT). The TyrA domain is predicted to belong to the TyrA_c_Δ _class when used as a query input into the AroPath Specificity Predictor Tool [40]. We speculated earlier that the •AroQ fusion domain of *Archaeoglobus *may exercise cooperative interactions with TyrA_c_Δ_, as appears to occur between the AroQ•TyrA_c_Δ _domains of *E. coli *and its relatives.

### *tyrA *in its syntenic context

Although the genes of prokaryotes have clearly been subject to frequent scrambling, some gene-gene associations persist more tenaciously than others. Xie et al. [5,6] asserted that one such ancestral gene string that has resisted scrambling forces is *hisH*_*b *_> *tyrA *> *aroF*. As suggested above, contemporary gene fusions can serve as frozen-in-time indicators of ancient gene organizations that were later obscured by gene-scrambling events. Another gene string that is often within the syntenic region of *hisH*_b_, *tyrA*, and *aroF *is *cmk *> *rpsA*. Gene synteny in prokaryotes has not been easily recognized because substantial manual scrutiny in combination with a sufficient density of genomic representation on a given portion of the phylogenetic tree is necessary to detect patterns of synteny that are camouflaged by frequent scrambling events (inversion, deletion and transposition).

**Figure 5:**
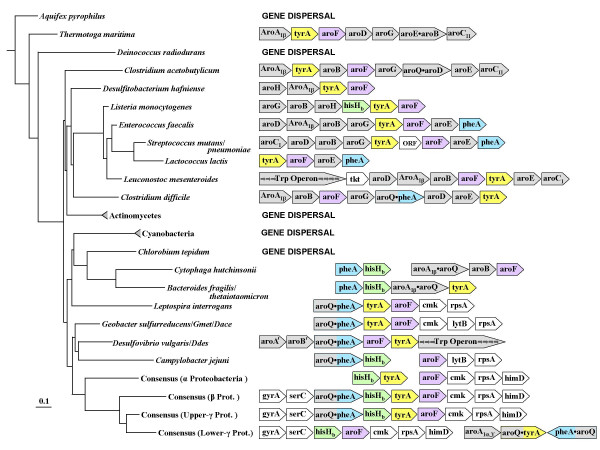
Context of gene organization for *tyrA*, profiled against the 16S rRNA tree of the domain *Bacteria*. *pheA*, *hisH*_b_, *tyrA*, and *aroF *are color coded. Lineages typified by complete dispersal of aromatic-pathway genes are indicated by "GENE DISPERSAL". Gmet refers to *Geobacter metallireducens*; Dace refers to *Desulfuromonas acetoxidans*; and Ddes refers to *Desulfovibrio desulfuricans*. Consensus gene organizations are shown for the alpha and beta divisions of the Proteobacteria. The gamma division is subdivided to yield consensus gene organizations for the upper- and lower-gamma (enteric lineage) organisms. Genes that are adjacent and share a common transcriptional direction appear to reside in operons (or supraoperons). Any white spacing indicates substantial separation of the gene clusters shown in the genome. Genes of special interest are color coded, other genes of aromatic biosynthesis are shown in gray and all other genes are shown in white.

The domain *Bacteria *is now represented by a collection of sequenced genomes that is progressively approaching the genomic densities needed for meaningful analysis. Figure 5 provides a visual sense of the frequency with which *tyrA *is closely positioned with other genes of aromatic biosynthesis, as well as the underlying patterns of overall synteny. These patterns are unstable, and yet persistent traces of synteny can be seen where genomic representation is sufficiently dense. The four genes of particular emphasis in this paper are color coded. Other genes that are engaged in aromatic biosynthesis are colored grey, and any other genes are white. At a very deep level of phylogenetic branching, *Thermotoga *exhibits a *tyrA *gene flanked by seven genes encoding all of the common steps of aromatic biosynthesis (two of them being fused). Since closely related genomes are not yet available here, we cannot judge whether these genes came together recently or whether an ancient pattern of synteny has been retained. Although *tyrA *is not linked to any functionally relevant genes in *Aquifex*, representing another point of deep phylogenetic branching, this does not necessarily mean that *tyrA *was not already generally associated with other aromatic-pathway genes at an early time. For reasons that are totally mysterious, certain scattered lineages exhibit a total lack of operon organization for aromatic-pathway genes (and indeed for most other biosynthetic pathways, such as that for histidine biosynthesis). These lineages (Fig. 5) include, besides *Aquifex*, those of *Deinococcus*, the actinomycetes, the cyanobacteria, and *Chlorobium*. Except for the actinomycetes, this phenomenon of total gene dispersal also applies to genes of tryptophan biosynthesis [7,8].

When the various examples of *hisH*_b _> *tyrA *> *aroF *linkage are mapped on a 16S rRNA tree, they first appear in gram-positive bacteria. In *Bacillus *and related organisms (such as *Listeria*), the *hisH*_*b *_> *tyrA *> *aroF *unit is associated with a large ancestral operon consisting of *aroG *> *aroB *> *aroH *> *hisH*_*b *_> *tyrA*_*p*_> *aroF*. *Bacillus *additionally possesses the *cmk *> *rpsA *unit, albeit in a separate location. Interestingly, in one narrow subclade (*B. subtilis*, *B. halodurans *and *B. stearothermophilus*) the *trp *operon has been inserted between *aroH *and *hisH*_*b *_to yield a supraoperon that has been fully characterized as a complex functional unit [51]. See Xie et al. [7] for a full presentation of evolutionary interpretation relevant to the latter. Though highly scrambled, a pattern of association of *pheA *with *hisH*_b _> *tyrA *>*aroF *is suggested by linkage patterns seen at the hierarchical level of C*ytophaga *and *Bacteroides *(Fig. 5). *aroQ *became associated with *pheA *through gene fusion as early as the divergence of the *Spirochaetes *to yield an *aroQ•pheA*>*tyrA*>*aroF*>*cmk*>*rpsA *linkage unit (*Leptospira interrogans *in Fig. 5). The *aroQ•pheA *gene associated with *tyrA *and *aroF *in *Clostridium difficile *appears to have arisen from a distinctly different fusion event than that present in delta, epsilon, beta and upper-gamma Proteobacteria or from that present in lower-gamma Proteobacteria (based upon analysis of inter-domain linker regions; unpublished data).

**Figure 6:**
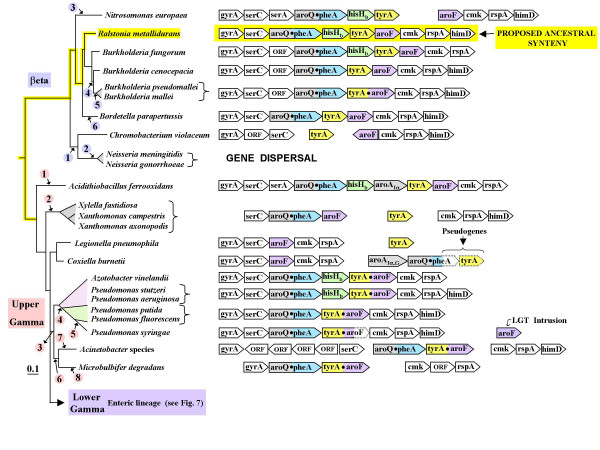
Zoom-in from Fig. 5 showing *tyrA *synteny for the beta Proteobacteria and the upper-gamma Proteobacteria. The tree shown, based upon 16S rRNA sequences of the indicated organisms, indicates correct branching orders, but (to facilitate presentation) is not strictly correct in proportion. Circled numbers (in violet) indicate deduced evolutionary events for the beta Proteobacteria (see top of Table 5), whereas circled numbers (in pink; see bottom of Table 5) correspond to deduced evolutionary events for the upper-gamma Proteobacteria. Gene organizations of organisms indicated are shown on the right. The dotted outlining of some gene boxes in *Coxiella burnetii *and in *P. syringae *indicates pseudogene status.

**Figure 7:**
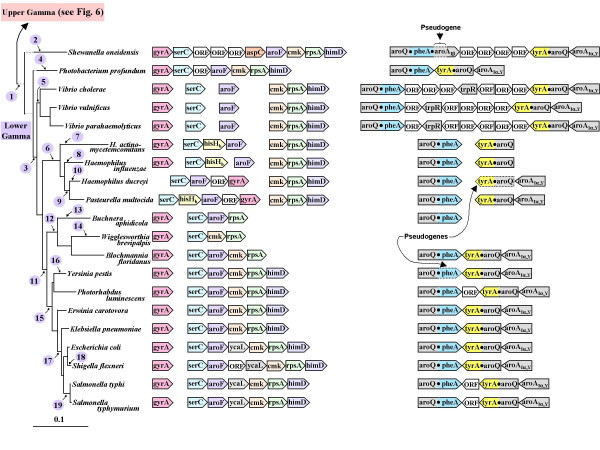
Zoom-in from Fig. 5 showing *tyrA *synteny for the lower-gamma Proteobacteria (enteric lineage). Deduced phylogenetic events numbered on the left are described in Table 6. The branching position for *Buchnera *is as suggested in ref. [7]. Dotted horizontal lines near the top of the tree indicate branch lengths that were shortened for convenience of presentation. Dotted outlining of boxes around some genes indicates their pseudogene status. It is unknown if the various open reading frame (ORF) insertions are functional.

Consensus ancestral gene organizations for the most densely represented divisions of Proteobacteria have been deduced as shown at the bottom of Fig. 5. Detailed information that supports a deduced consensus for ancestral gene organizations with respect to beta Proteobacteria, upper-gamma Proteobacteria, and lower-gamma Proteobacteria are shown later (Figs. 6, 7). We suggest that the last common ancestor of all Proteobacteria possessed the gene organization *aroQ•pheA*>*hisH*_b_>*tyrA*>*aroF*>*cmk*>*rpsA*. This is similar to the synteny that has been retained in general by the beta Proteobacteria and the upper-gamma Proteobacteria. The *aroQ•pheA*>*hisH*_b_>*tyrA *portion likely specified all the catalytic requirements for conversion of chorismate to PHE and conversion of chorismate to TYR. Chorismate mutase activity specified by the *aroQ *domain could supply PPA for both PHE and TYR biosynthesis. Likewise, HisH_b_, widely utilized as an aromatic aminotransferase [49], could also function for both PHE and TYR biosynthesis. Though currently available members of delta and epsilon Proteobacteria exhibit substantial gene scrambling, the various fragmentary linkage patterns seen provide support for the ancestor proposed. *Geobacter *(and other Delta_1 members) has the *aroQ•pheA *> *tyrA *> *aroF *> *cmk *> *rpsA *linkage group (with *lytB *inserted between *cmk *and *rpsA*). *Desulfovibrio vulgaris*, another delta Proteobacterium (Delta_2) that is highly divergent from *Geobacter*, has a very interesting pattern of conservation and scrambling. *aroQ•pheA *> *aroF *> *tyrA *has been attached to a complete 7-gene *trp *operon. *hisH*_b _> *cmk *(not shown in Fig. 5) is completely separated from *rpsA*. The supraoperonic gene organization shown for *D. vulgaris *begins with two recently discovered genes, herein denoted *aroA*' and *aroB*', that encode enzymes specifying an alternative biochemical route to dehydroquinate [52]. The epsilon Proteobacteria all display significant gene scrambling, but piecemeal evidence for the unscrambled ancestor proposed is present. For example, *Campylobacter jejuni *possesses an *aroQ•pheA *> *hisH*_b _unit, as well as *aroF > lytB > rpsA *(Fig. 5). *Wollinella succinogenes *and *Helicobacter hepaticus *both possesses an *aroF *> *lytB *> *rpsA *unit.

The ancestor of alpha Proteobacteria has lost the *aroQ•pheA *fusion, and a stand-alone *pheA *is consistently observed. Members of this group are quite uniform in the stable possession of *hisH*_b _> *tyrA *and *aroF *> *cmk *> *rpsA *as two separated linkage groups. The beta Proteobacteria are represented by members that have the gene organization: *serC *> *aroQ•pheA *> *hisH*_b _> *tyrA *> *aroF *> *cmk *> *rpsA*. This is also seen in the members of the upper-gamma Proteobacteria.

Figure 5 includes organisms that illustrate the traces of synteny that can be detected in *Bacteria *where overall genome representation is just barely adequate. The following two figures illustrate how syntenic patterns of more resolution and refinement become evident with denser genome representation.

### Zooming in on syntenic contexts of proteobacteria

#### Beta proteobacteria and upper-gamma proteobacteria

The beta Proteobacteria exhibit a dynamic but still interpretable pattern of altered synteny (Fig. 6 and Table 5). Species of *Ralstonia *have retained the proposed ancestral synteny that is marked with yellow highlighting in Fig. 6. This syntenic organization is such that the aromatic-gene unit *aroQ•pheA *> *hisH*_b _> *tyrA *> *aroF *is nested between *gyrA *> *serC *at the leftward flank and *cmk *> *rpsA *> *himD *at the rightward flank. Species of *Burkholderia *(the next closest lineage) are almost identical, but exhibit individual evolutionary events (marked by circled numbers on the left, which correspond to a description of the proposed evolutionary events given in companion Table 5). These events include gene insertion, loss of *hisH*_b_, translocation of genes away from the ancestral supraoperon, and fusion of *tyrA *and *aroF *(in the common ancestor of *B. mallei *and *B. pseudomallei*). At a deeper level in the beta Proteobacteria section of the tree, *Nitrosomonas europaea *exhibits a separation of the ancestral supraoperon between *tyrA *and *aroF*. Either a very large insertion was made between *tyrA *and *aroF*, or one of the two gene clusters shown was transposed as part of a sufficiently large segment to include all of the conserved flanking genes. In *Chromobacterium violaceum tyrA *has become completely isolated from other gene members of the ancestral supraoperon, and *aroF *has assumed an inverted orientation with respect to *cmk*. Species of *Neisseria *exhibit no remnants of supraoperon synteny at all, and wholesale dispersal of all the supraoperon genes has occurred. (It is interesting that among the beta Proteobacteria, *Neisseria *species are also unique in that all of the *trp*-pathway genes are dispersed [7]).

The gamma Proteobacteria have separated into two distinctly different synteny patterns. The lower-gamma Proteobacteria have undergone marked syntenic change (see below). The assemblage portrayed between *Acidithiobacillus *and *Microbulbifer *in the lower part of Fig. 6 (termed the upper-gamma Proteobacteria) exhibit a strong overall syntenic resemblance of supraoperon genes to that of the beta Proteobacteria. *Acidithiobacillus *possesses a near-intact ancestral supraoperon, differing only in having two insertions: one gene encoding 3-deoxy-D-**arabino**-heptulosonate 7-phosphate (DAHP) synthase between *hisH*_*b *_and *tyrA*, and the other being the insertion of *serA *between *serC *and *aroQ•pheA*. *Pseudomonas aeruginosa *and *P. stutzeri *have also retained nearly intact ancestral supraoperons, differing only in the fusion of *tyrA *and aroF. The *serC *> *aroQ•pheA *> *hisH*_b _> *tyrA•aroF *> *cmk *> *rpsA *supraoperon has been studied in *P. stutzeri *[5,6]. The *tyrA•aroF *fusion occurred in the common ancestor of the clade shown between *Azotobacter *and *Microbulbifer *in Fig. 6. The supraoperons of *P. syringae, P. fluorescens *and *P. putida *lack *hisH*_b_. *P. syringae *exhibits a recent C-terminal truncation of the *aroF *domain, coupled with acquisition elsewhere in the genome of a free-standing •*aroF *that is not phylogenetically congruent (probably of LGT origin). *Acinetobacter *sp. and *Microbulbifer degradans *possess an *aroQ•pheA *> *tyrA•aroF *unit that has become dissociated from *serC *at one end and from *cmk *on the other end. In *Xylella *and *Xanthomonas*, *hisH*_*b *_has been deleted from the genome and *tyrA *has been transposed away from *serC *> *aroQ•pheA *> *aroF*. The latter unit has been transposed away from *gyrA*, the ancestral flanking gene. On the other hand, *cmk *> *rpsA *has remained next to *himD*, the gene usually flanking *rpsA*.

#### The enteric lineage

The lower-gamma Proteobacteria differ sharply from upper-gamma Proteobacteria in their possession of the *tyrA*_*c_Δ *_class of *tyrA *and its fusion with *aroQ*. In Fig. 2 this clade of AroQ•TyrA_c_Δ _fusions was presented as one exhibiting absolute specificity for NAD^+^, combined with an overwhelming but not complete specificity for PPA. In Fig. 7 the gene synteny associated with *tyrA*_*c_Δ *_is profiled against the 16S rRNA phylogenetic trees of the lower-gamma Proteobacteria possessing these genes, and the proposed evolutionary events are summarized in the companion Table 6. Figure 5 has indicated a synteny consensus for the common ancestor at this hierarchical level whereby *gyrA *> *serC *> *hisH*_b _> *aroF *> *cmk *> *rpsA *parallels the ancestral synteny of β-Proteobacteria, but without *aroQ•pheA *or *tyrA *in the middle of the linkage group. Many dynamic evolutionary events of altered aromatic biosynthesis have occurred within the lower-gamma Proteobacteria since their divergence from the upper-gamma Proteobacteria. This includes the emergence of three allosterically distinct DAHP synthases, one of which now comprises the two-gene, three-domain *tyr *operon (*aroA*_Iα_*Y *_> *aroQ•tyrA*_*c_Δ*_). The upper-gamma Proteobacteria characteristically possess the *aroA*_*Iα *_paralogs encoding AroA_Iα_H _(TRP-inhibited DAHP synthase) and AroA_Iα_Y _(TYR-inhibited DAHP synthase). It has been asserted that AroA_Iα_F _(PHE-inhibited DAHP synthase) was the most recent paralog, acquired just after divergence of the lower-gamma Proteobacteria [53]. It is bizarre that *Shewanella oneidensis *possesses a pseudogene of *aroA*_Iβ _fused to the C terminus of *aroQ•pheA*. The *aroA*_Iβ _subclass of Family-I DAHP synthases is not usually observed in gram-negative bacteria [54].

The dissociation of *tyrA*_*c_Δ *_from the *serC*/*rpsA *linkage group correlates with the fusion of *aroQ *with *tyrA*_*c_Δ*_. The *aroQ•pheA *fusion has also escaped from the ser*C*/*rpsA *linkage grouping and has become linked with the newly emerged *tyr *operon. Some sort of duplication and recombinational event between *aroQ•pheA *and *tyrA*_*c_Δ *_may have led to the creation of *aroQ•tyrA*_*c_Δ *_since the AroQ•PheA proteins of lower-gamma Proteobacteria are distinct from AroQ•PheA proteins of other Proteobacteria with respect to the inter-domain linker length and the indel content (data not shown).

Although it usually is absent from the lower-gamma Proteobacteria, HisH_b _has persisted as the broad-specificity aromatic aminotransferase in the *Pasteurella/Haemophilus *grouping where two *hisH *paralogs are generally present, one of narrow specificity (denoted *hisH*_n_) being within the histidine operon. The *aspC *gene next to *aroF *in *Shewanella *is a paralog that probably functions as an aromatic aminotransferase, suggestive of the situation in the *E. coli *grouping where *tyrB *is a close paralog relative of *aspC*, tyrB having become specialized for aromatic biosynthesis [49]. Gene reduction associated with both endosymbiotic and pathogenic lifestyles are evident. Thus, *Buchnera *lacks *tyrA*, *cmk*, *hisH*, *tyrB*, and possesses only a single *aroA*_*Iα *_species (*aroA*_Iα_*H*_). *Haemophilus ducreyi *also lacks *tyrA*, as well as aroA_Iα_H _and the entire *trp *operon [5].

### TyrA in its context of regulation

#### TyrR regulon

**Figure 8:**
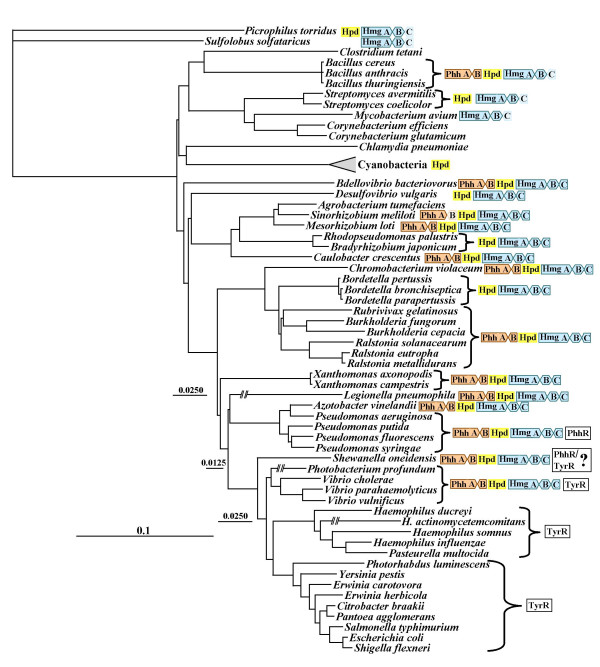
Distribution of modules of aromatic catabolism mapped on a 16S rRNA tree. In this figure, only presence or absence (not gene order) is indicated. The Phh module (orange) consists of phenylalanine hydroxylase (PhhA), carbinolamine dehydratase (PhhB), and tyrosine aminotransferase (not shown, see Text), and accomplishes the overall conversion of PHE to 4-hydroxyphenylpyruvate. The Hpd module (yellow) is 4-hydroxyphenylpyruvate dioxygenase, which converts 4-hydroxyphenylpyruvate to homogentisate. The Hmg module (blue) catalyzes the 3-step conversion of homogentisate to acetoacetate and fumarate. The distribution of PhhR and TyrR is shown in boxes. In some cases the HmgC member is shaded light blue to indicate that the gene encoding this isomerase could not be found and is probably encoded by an as yet unknown analog. Some long branches are drawn with gaps that represent 25% of the length of the scale bar.

Knowledge of the gene regulation impacting TyrA in prokaryotes is sparse, being limited to the lower-gamma Proteobacteria. Here, extensive information gathered from *E. coli *has revealed that *aroQ•tyrA*_*c_Δ *_belongs to a large regulon controlled by the TyrR repressor. The limited phylogenetic distribution of TyrR, being present only in the lower-gamma Proteobacteria (Fig. 8), indicates that it is a recent evolutionary acquisition. In *E. coli *the regulon members that are under the control of *tyrR *are the *aroA*_Iα_Y _> *tyrA *operon, the *aroLM *operon, *tyrP*, *tyrB*, *aroP*, *mtr*, *aroA*_Iα_F _and *tyrR *itself [55]. Thus, *tyrR *not only regulates the tyrosine branch of the pathway, but heavily impacts the common pathway and the transport of all three aromatic amino acids as well.

Although outside the scope of this study, a logical expansion of it would be to examine the individual evolutionary histories of all the members of the contemporary *E. coli *TyrR regulon, i.e., asking when and in what order did these genes come under the influence of *tyrR*? Clearly, the recruitment of structural genes by *tyrR *has been recent, quite dynamic and even now, exhibits evidence of further ongoing change. For example, tyrosine phenol-lyase (a catabolic enzyme that is only sparsely present in gamma Proteobacteria) has been recruited to the TyrR regulons of *Erwinia herbicola *[56] and *Citrobacter freundii *[57]. In these cases, not only does TyrR perform as a transcriptional activator, but it requires cyclic AMP receptor protein and integration host factor to do so.

As exemplified by *E. coli*, TyrR is generally a repressor. However, the transcriptional expression of *mtr *is activated by TyrR in the presence of TYR, and *tyrP *is activated in the presence of PHE (although it is repressed in the presence of TYR). The N-terminal domain of TyrR has been associated with the ability of TyrR to activate transcription in the case of *mtr *and *tyrP *[55]. Members of the *Haemophilus/Pasteurella *lineage have all lost the N-terminal domain and presumably all lack the ability to accomplish transcriptional activation, as has been demonstrated experimentally with *H. influenzae *TyrR [58].

In view of the interesting complexity that two operons (*mtr *and *aroLM*) in *E. coli *are regulated by both *tyrR *and *trpR *[55], it may be more than coincidental that *tyrR *and *trpR *seem to have emerged at about the same evolutionary time, i.e., coincident with the divergence of the upper-gamma Proteobacteria from the lower-gamma Proteobacteria (Fig. 7). A possible interaction between the TyrR and TrpR proteins has been noted [55].

#### PhhR in relationship to aromatic catabolism

Arias-Barrau et al. [59] have recently characterized a central catabolic pathway (Hmg) that degrades homogentisate in three steps to fumarate and acetoacetate as a source of carbon and energy. One of several peripheral pathways feeding into the central pathway begins with PHE and produces homogentisate via the reactions of phenylalanine hydroxylase (Phh), aromatic aminotransferase, and 4-hydroxyphenylpyruvate dioxygenase (Hpd). In the absence of Phh, a shorter version of the peripheral pathway is one that can use TYR, but not PHE, as a source of carbon and energy. In Fig. 8 the presence of Phh, Hpd, and Hmg segments of catabolism are mapped on a 16S rRNA tree. (The aromatic aminotransferase distribution is not shown since a multiplicity of aromatic aminotransferases having overlapping substrate specificities makes it particularly challenging to identify the functional role [49].) The cyanobacteria are unique among *Bacteria *in the use of Hpd for a completely different metabolic role unrelated to aromatic catabolism, i.e., the synthesis of vitamin E derivatives [60].

**Figure 9:**
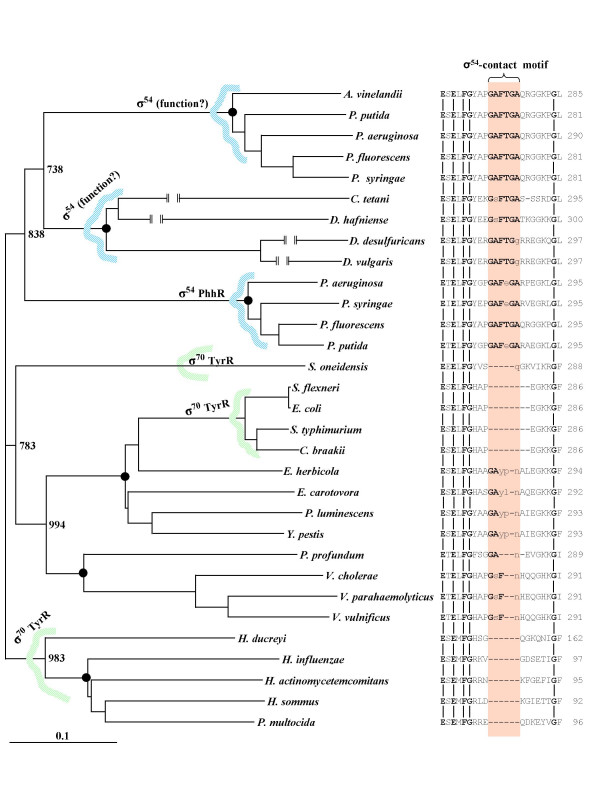
Protein tree of TyrR homologs. Nodes supported by bootstrap values of 998 or more are marked with solid circles, and the bootstrap values for nodes internal to these are shown. Generic names relevant to the organism abbreviations can be viewed in Fig. 8. A conserved region containing the **σ**^54 ^contact motif **GAFTGA **is highlighted as an orange band. "Imperfect" residues in this region are shown in lower-case fonts. Residue numbers are shown at the right. TyrR and PhhR are regulators of **σ**^70 ^and **σ**^54 ^promoters, respectively. Four **σ**^54 ^proteins of unknown function have very long branches, and to facilitate the visual presentation, the gaps in branch continuity shown represent a scale-bar distance of 0.1. Clades possessing **σ**^54 ^regulators are indicated with blue stripes, and **σ**^70 ^regulators are indicated with green stripes.

PhhR is a homolog of TyrR that has been shown in *P. aeruginosa *to be a divergently transcribed activator of a 3-gene operon needed for PHE and TYR catabolism [61]. The structural genes encode phenylalanine hydroxylase (*phhA*), carbinolamine dehydratase (*phhB*) and 4-hydroxyphenylpyruvate aminotransferase (*phhC*), and are powered by a σ^54 ^promoter [61,62]. PhhR evolved relatively recently since it is only present in some gamma Proteobacteria (Fig. 8). The ancestral regulatory gene for the Phh peripheral pathway may have been a member of the leucine-responsive regulatory protein/asparagine synthase C (Lrp/AsnC) family judging from the adjacent and divergently oriented position of *asnC *genes to *phhA *in organisms such as *Xanthomonas axonopodis *and *Mesorhizobium loti*. A recent overview of the many different regulator families involved in the control of aromatic catabolism conveys an emerging sense of the variety and dynamic evolutionary processes that underlie aromatic catabolism [63]. Occasional distant homologs of *phhR *that appear in erratic fashion (see Fig. 9) may have some other regulatory function. For example, *Clostridium tetani *may use its PhhR homolog as a transcriptional activator of the gene encoding tyrosine phenol-lyase, as occurs in species of *Erwinia *[56] and Citrobacter [57].

#### Relationship of TyrR and PhhR

What might be of origin of TyrR? TyrR is an anomalous member of the large prokaryote family of σ^54 ^enhancer-binding proteins that activate promoters dependent upon σ^54^. TyrR is unique within its homology grouping in that it targets σ^70 ^promoters for regulation, usually (but not always) being a repressor. Its closest homolog relative is PhhR, a canonical member of σ^54 ^enhancer-binding proteins. σ^54^-dependent enhancer proteins possess a highly conserved σ^54^-contact motif, GAFTGA, that is intimately involved in formation of the ternary complex of enhancer and σ^54^-RNA polymerase holoenzyme [64]. This is perfectly or nearly perfectly retained in the upper clades shown in Fig. 9, but is disrupted or completely absent in the clades between *Shewanella oneidensis *and *Pasteurella multocida*. The deeper phylogenetic distribution of PhhR (Fig. 8) suggests that TyrR evolved as a variant of PhhR. If correct, a regulatory gene that is oriented to catabolism (*phhR*), and itself of relatively recent origin, was conscripted even more recently for a completely new role in the regulation of primary biosynthesis (*tyrR*).

Consistent with the latter supposition, the gain of TyrR generally correlates with the loss of competence for aromatic catabolism (Fig. 8). In contrast to the *Citrobacter*/*Salmonella*/*Escherichia*/*Shigella *and the *Pasteurella*/*Haemophilus *clades (whose TyrR homologs completely lack the GAFTGA motif), the remaining enteric clades have retained some residues in this region. These residues appear to be more than random remnants. It would be interesting to know if these residues have any functional significance. Indeed, the *Photobacterium/Vibrio *clade has retained the ancestral catabolic capabilities (Fig. 8) that would appear to demand retention of regulation via PhhR; yet the parallelism of the overall features of biosynthesis that are shared with other lower-gamma Proteobacteria would seem, on the other hand, to demand TyrR-mediated regulation. Perhaps this "TyrR" species participates in the regulation of both catabolic and biosynthetic genes. In this connection, it is interesting that Chaney et al. [64] found that a change in the GAFTGA motif of NifA could be partially "suppressed" by mutational changes in the N-terminal region of σ^54^.

Even more striking as a possible evolutionary intermediate is the most outlying member of the lower- gamma Proteobacteria, *Shewanella oneidensis*. The position of its TyrR on the protein tree parallels expectations based on the 16S rRNA tree. This, plus the conservation of the TyrR regulon features and the overall gene synteny suggest *E. coli*-like function as TyrR, i.e. acting as a general repressor of regulon-member σ^70 ^promoters engaged in aromatic biosynthesis. However, the location of "*tyrR*" in *S. oneidensis *between *phhA *and *phhB *on one side, and *hmgB *and *hmgC *on the other side, strongly implies some kind of regulatory relationship with the catabolic genes. It would be quite interesting to determine experimentally whether "TyrR" in *S. oneidensis *(and maybe *Vibrio*, as well) can function as a repressor of the usual suite of σ^70 ^promoters, as well as an activator of σ^54 ^promoters for *phhA*/*phhB *and/or *hmgB*/*hmgC*.

We suggest that TyrR evolved as a modified version of PhhR as follows. In view of the distribution of genes encoding PhhR and TyrR, as well as the aforementioned catabolic enzymes, the most parsimonious evolutionary scenario may be that central and peripheral catabolic pathways depicted in Fig. 8 are quite ancient, but acquisition of PhhR as a σ^54^-dependent activator of phenylalanine hydroxylase was quite recent, originating about the time of divergence of gamma Proteobacteria. The clade defined by *Shewanella/Vibrio/Photobacterium *retained the catabolic pathway, whereas the other enteric lineages discarded the catabolic pathway, but retained PhhR, which was then recruited as a σ^70^-dependent regulator of aromatic biosynthesis (TyrR).

#### Regulation by attenuation

A widespread mechanism of regulation is via an attenuation mechanism whereby transcripts initiated at given promoters can be terminated prior to reaching the structural genes of an operon. Whether termination occurs usually depends on the balance (dictated by a variety of mechanisms) between mutually exclusive terminator and anti-terminator structures [65].

Merino has developed a website [66] to provide a database of putative attenuators ahead of various operons in *Bacteria*. We screened this database for likely attenuators relevant to the regulation of *tyrA*. Table 7 shows intriguing results that point to significant experimental work that would be desirable. *tyrA *is frequently a member of apparent supraoperons, as alluded to elsewhere in this paper, and some of these appear to be large gene clusters controlled by attenuation. Substantial work is needed to establish the depth of clades possessing a given attenuator. For example, the *hisH*_*b *_> *tyrA *operon is reliably present throughout all alpha Proteobacteria. Since *Agrobacterium tumefaciens *has been found to possess an attenuator ahead of the *hisH*_*b *_> *tyrA *operon, one might reasonably expect that most of the alpha Proteobacteria would possess the attenuator as well. If not, this attenuator would have been a very recent evolutionary innovation. Likewise, since the *aroA*_Iα_Y _> *tyrA *operon is widely present throughout the lower-gamma Proteobacteria, it would be interesting to confirm whether only the several species of *Vibrio *identified on the Merino website have an attenuator ahead of this operon (or whether other attenuators present are too weak to exceed the threshold imposed for preliminary detection).

Some of the supraoperons that appear to be controlled by attenuation are interesting in that they contain the majority of genes needed for both PHE and TYR biosynthesis, e.g., the supraoperons in *Enterococcus faecalis *and *Streptococcus pneumoniae*. The latter organism displays two attenuator units. The supraoperon of *Desulfovibrio vulgaris *is novel in that it begins with two relatively rare genes encoding alternative enzyme steps for aromatic biosynthesis [52], denoted here as *aroA*' and *aroB*'. The leading five genes are adjacent to the seven-gene *trp *operon.

## Conclusion

Protein divergence within a vertical genealogy is not necessarily smooth and progressive. Qualitative biochemical innovations can result in a barrage of new selective pressures that result in evolutionary jumps. The consequent incongruence might easily be mistaken for LGT. The basis for evolutionary jumps will usually only be recognized by detailed and comprehensive analyses of any given subsystem. Examples in this study are as follows. (**i**) The *tyrA*_c_Δ _gene of the lower-gamma Proteobacteria has diverged markedly from *tyrA*_c _of the upper-gamma Proteobacteria. Here the milestone event was fusion of *aroQ *to a putative *tyrA*_c _in the ancestor of lower-gamma Proteobacteria to produce *aroQ•tyrA*_c_Δ_. Indels within the •*tyrA*_c_Δ _domain presumably reflect a multiplicity of selections for functional interactions known to exist between the two fused domains as discussed earlier. (**ii**) Members of the subclass taxon *Actinobacteridae *possess TyrA_a _proteins that separate into two distinct groupings. The presumed ancestral _NAD(p)_TyrA_a _that is still present in the *Actinobacteridae*_1 clade very likely spawned the divergent NAD^+^-specific variety of TyrA_a _to yield the contemporary *Actinobacteridae*_2 clade.

The previous evolutionary analysis of *trp-*pathway genes [7,8] can be viewed as a model for comparable studies with other gene systems. Expansion to the greater aromatic pathway is a logical extension. The dynamics of evolutionary change for *tyrA *can be matched to the dynamics exhibited by the *trp *system. For example, the lower-gamma Proteobacteria separate as a distinct phylogenetic unit from beta Proteobacteria and upper-gamma Proteobacteria on criteria defined by milestone evolutionary events that altered many character states of both tryptophan and tyrosine biosynthesis in the lower-gamma Proteobacteria. In the future one can anticipate that comprehensive and systematic phylogenetic analysis of each protein member of the TYR, PHE and TRP branches, the common aromatic-pathway trunk, and minor vitamin-like branches (such as the 4-aminobenzoate/folate branch) will accommodate a progressively integrated picture of the entire aromatic network, including catabolic pathways and many other specialized pathways.

## Methods

### TyrA sequences

Most TyrA sequences were obtained from the National Center for Biotechnology Information (NCBI) [16]. TyrA sequences from incomplete genomes were retrieved from the PEDANT database [67]. Several sequences in our curated TyrA collection have been corrected for incorrect translation start sites. Various curated TyrA sequence files can be downloaded from our website. These files include complete sequences, trimmed catalytic-core domains, and amino-acid sequence segments that are relevant to specificity for pyridine nucleotide or to specificity for the cyclohexadienyl substrate. The sequence files are summarized in Table 3.

### Congruency groupings

TyrA proteins that cluster together on the TyrA protein tree in congruence with the 16S rRNA tree are called congruency groups. Exact correspondence of branching orders is not necessarily observed. So far, congruency groupings have been assembled for tryptophan-protein concatenates [8] and for TyrA proteins. Completion of equivalent work with the remaining aromatic-pathway segments will identify the repertoire of bacterial organisms in possession of a "pure" vertical genealogy with respect to aromatic biosynthesis. Congruency groups for TyrA can be accessed at our AroPath website [9], where a listing of the membership of congruency groups is maintained and updated. Any members of congruency-group clusters, whose position there is incongruent with 16S rRNA expectations, probably (but not necessarily) originated by LGT. The donor lineage may not be obvious, but as more genomes come on line, many cases where donor identities are currently unknown may become revealed. A listing of "orphan" TyrA proteins that belong to no current congruency group is given. Such orphans reflect the lack of sufficient genome representation in particular phylogenetic regions and undoubtedly will become the nucleus for additional congruency groups in due course.

### Alignments

Multiple alignments were obtained by use of the ClustalW or ClustalX programs (Version 1.83) [68]. Manual adjustments were needed in the region of the GxGxxG motif for binding of pyridine nucleotide cofactor in the N-terminal region of TyrA proteins. Guidance for alignment was assisted by maximizing conformation with the Wierenga fingerprint, making allowance for a variable loop of 2–5 residues [26]. This was done with the assistance of the BioEdit multiple alignment tool of Hall (5.0.9 Edition) [69]. The refined multiple alignment was used as input for generation of a phylogenetic tree using the phylogeny inference package (Version 3.2), PHYLIP [70]. The neighbor-joining program was used to obtain a distance-based tree. The distance matrix was obtained by use of Protdist with a Dayhoff Pam matrix. The Seqboot and Consense programs were then applied to assess the statistical support of the tree using bootstrap resampling (1,000 replications). We also used the ANCESCON package [71], which produced similar results as shown in Fig. 2 (albeit with even wider separation of many groups). The presence of regulatory domains (ACT and REG) was accepted when indicated by the Domain Architecture Retrieval Tool (DART) on the BLAST menu at NCBI [16].

### Profile HMMs

Profile hidden Markov models for each of the four TyrA subfamilies, TyrA_a_, TyrA_c_, TyrA_p _and *tyrA*_*c_Δ*_, were built using Sean Eddy's HMMER package [72]. The HMMs were generated from our file of curated cyclohexadienyl-substrate core segments (see Table 3). The seed sequences for each subfamily were first aligned using ClustalW [68]. The resulting multiple sequence alignments were then manually edited to produce more accurate alignment of the seed sequences. Finally, the edited multiple sequence alignments were used to generate the profile HMMs for each TyrA subfamily.

### Appraisal of gene fusions as one-time or multiple events

Whether any given contemporary gene fusions tracked back to a fusion event in a common ancestor or whether they occurred independently was evaluated by phylogenetic analysis of the individual protein domains and by inspection of the inter-domain linker region. Linker regions were determined by multiple alignments of fusion sequences with corresponding free-standing domains present in the closest relatives to organisms that lack the gene fusions.

## Authors' contributions

JS and MW integrated this specific effort with the broader and ongoing objective of implementing a dynamic and progressively updateable website (AroPath). JS also made substantial contributions to the bioinformatic work. CB did all of the art work and a majority of the bioinformatic analyses. RJ provided initial guiding concepts, a general organizational overview, and assembled the initial manuscript draft. CB, RJ, and JS contributed to the formulation of conclusions made, and all of the authors read and approved the final version of the manuscript.

## Supplementary Material

Additional File 1 Table S1, entitled "Key to organism acronyms and sequence identifiers", is provided as supplementary material in an html document. This table contains the full collection of sequence data and annotations contained in this paper, and gene identification (gi) numbers are included and hyperlinked to facilitate access to the corresponding GenBank records. For future reference to a progressively updated table, refer to the AroPath website [73]. Click here for file

## Figures and Tables

**Table 1:** Abbreviations used to designate substrate specificities of *tyrA/*TyrA homologs

Abbreviation^a^		
Gene	Gene Product	Description of specificity^b^

*tyrA*_x_	TyrA_x_	Specificity for cyclohexadienyl substrate is unknown
*tyrA*_c_	TyrA_c_	Broad-specificity cyclohexadienyl dehydrogenase (CDH)
*tyrA*_p_	TyrA_p_	Narrow-specificity prephenate dehydrogenase (PDH)
TyrA_c_Δ_	TyrA_c_Δ_	Broad-specificity cyclohexadienyl dehydrogenase having catalytic-core indels in correlation with an extra-core extension
*tyrA*_a_	TyrA_a_	Narrow-specificity arogenate dehydrogenase (ADH)
_*NAD*_*tyrA*_a_	_NAD_TyrA_a_	TyrA homolog is AGN-specific and NAD^+^-specific
_*NADP*_*tyrA*_a_	_NADP_TyrA_a_	TyrA homolog is AGN-specific and NADP^+^-specific
_*NAD*(*P*)_*tyrA*_*a*_	_NAD(P)_TyrA_a_	TyrA homolog is AGN-specific but utilizes either NAD^+ ^or NADP^+^
_x_*tyrA*_x_	_x_TyrA_x_	Specificity for both the cyclohexadienyl and pyridine nucleotide substrates is unknown

^a^Abbreviations in the upper-table (upper 5 rows) indicate the specificities for the cyclohexadienyl substrate. Abbreviations in the lower-table (lower 4 rows) indicate specificities for both cyclohexadienyl (right subscripts) and pyridine nucleotide substrates (left subscripts). Combinations not shown can be deduced from the examples given, e.g., a TyrA homolog specific for prephenate and NAD^+ ^would be designated _NAD_TyrA_p_.

^b^The abbreviations CDH, PDH, and ADH (shown parenthetically) have been used frequently in the literature.

**Table 2:** Key to organism acronyms

**Organism**	**Abbreviation ** **in Paper**	**Abbreviation** **on website^b^**
*Acidithiobacillus ferrooxidans *ATCC 23270	Aferr	
*Acinetobacter *sp. ADP1	ACIN	
*Actinobacillus actinomycetemcomitans *HK1651	Aact	
*Actinomyces naeslundii *MG1	Anae	
*Actinoplanes teichomyceticus*	Atei	
*Agrobacterium tumefaciens *strain C58	Atum	
*Amycolatopsis balhimycina*	Abal	
*Amycolatopsis orientalis*	Aori	
*Anabaena *sp. PCC 7120	ANAB	
*Arabidopsis thaliana*	Atha	
*Archaeoglobus fulgidus *DSM 4304	Aful	Aful_1
*Azotobacter vinelandii*	Avin	Avin_1
*Bacillis anthracis *str. A2012	Bant	
*Bacillus cereus *ATCC 14579	Bcer	
*Bacillus halodurans *C-125	Bhal	Bhal_2
*Bacillus stearothermophilus*	Bste	
*Bacillis subtilis*	Bsub	
*Bacillus thuringiensis *israelensis	Bthu	
*Bifidobacterium longum *NCC2705	Blon	Blon_1
*Blochmannia floridanus*	Bflo	
*Bordetella bronchisepticus*	Bbro	
*Burkholderia cepacia *J2315	Bcep	
*Burkholderia fungorum *LB400	Bfun	
*Burkholderia mallei *ATCC 23344	Bmal	
*Burkholderia pseudomallei *K96243	Bpse	Bpse_6
*Campylobacter jejuni*	Cjej	
*Chromobacterium violaceum *ATCC 12472	Cvio	
*Corynebacterium diphtheriae *NCTC 13129	Cdip	
*Corynebacterium efficiens *YS-314	Ceff	
*Corynebacterium glutamicum *ATCC 13032	Cglu	Cglu_1
*Desulfovibrio desulfuricans *G20	Ddes	
*Desulfovibrio vulgaris *subsp. *vulgaris *strain Hildenborough	Dvul	
*Desulfuromonas acetoxidans*	Dace	Dace_5
*Enterococcus faecalis *V583	Efae_2	
*Enterococcus faecium*	Efae_1	Efae_1
*Erwinia carotovoa *subsp.*atroseptica *SCRI1043	Ecar	
*Escherichia coli *K12	Ecol	
*Geobacter metallireducens *GS-15	Gmet	
*Geobacter sulfurreducens *PCA	Gsul	
*Gloeobacter violaceus *PCC 7421	Gvio	
*Haemophilus influenzae *Rd KW20	Hinf	
*Helicobacter hepaticus *ATCC 51449	Hhep	
*Helicobacter pylori *26695	Hpyl	
*Klebsiella pneumoniae *subsp. *pneumoniae *MGH 78578	Kpne	
*Leifsonia xyli *subsp. *Xyli *strain CTCB07	Lxyl	
*Listeria innocua *Clip 11262	Linn	
*Listeria monocytogenes *EGD-e	Lmon	
*Lotus corniculatus *var. *japonicus*	Lcor	Lcor_3
*Lycopersicon esculentum*	Lesc	
*Methanococcus jannaschii*	Mjan	
*Methanopyrus kandleri *AV19	Mkan	Mkan_1
*Methanosarcina barkeri *strain Fusaro	Mbar	
*Methanothermobacter thermoautotrophicus *strain Delta H	Mthe	Mthe_7
*Microbulbifer degradans *2–40	Mdeg	
*Mycobacterium avium *subsp. *paratuberculosis *strain k10	Mavi	
*Mycobacterium bovis *TrEMBL	Mbov	Mbov_2
*Mycobacterium leprae *TN	Mlep	
*Mycobacterium tuberculosis *CDC1551	Mtub	
*Myxococcus xanthus *DK 1622	Mxan	
*Neisseria gonorrhoeae *FA 1090	Ngon	
*Nitrosomonas europaea *ATCC 19718	Neur	
*Nocardia farcinica *IFM 10152	Nfar	
*Nonomuraea *sp.	NONO	
*Nostoc punctiforme *PCC73102	Npun	Npun_1
*Novosphingomonas aromaticivorans *DSM 12444	Naro	
*Oceanobacillus iheyensis *THE831	Oihe	
*Oryza sativa *ssp. *japonica*	Osat	
*Pantoea agglomerans*	Pagg	
*Pasteurella multocida *subsp. *multocida *strain Pm70	Pmul	
*Photorhabdus luminescens *subsp. *laumondii *TT01	Plum	
*Prochlorococcus marinus *subsp. *pastoris *strain CCMP1378	Pmar_1	Pmar_3
*Prochlorococcus marinus *MIT9313	Pmar_2	Pmar_10
*Propionibacterium acnes *KPA171202	Pacn	
*Pseudomonas aeruginosa *PAO1	Paer	Paer_1
*Pseudomonas fluorescens *PfO-1	Pflu	
*Pseudomonas putida *KT2440	Pput	
*Pseudomonas stutzeri*	Pstu	
*Ralstonia eutropha *JMP134	Reut	
*Ralstonia solanacearum *GMI1000	Rsol	
*Rhodobacter capsulatus*	Rcap	
*Rhodobacter sphaeroides *2.4.1	Rsph	
*Rhodopseudomonas palustris *CGA009	Rpal	
*Rhodospirillum rubrum*	Rrub	Rrub_1
*Rubrobacter xylanophilus *DSM 9941	Rxyl	
*Saccharomyces cerevisiae*	Scer	
*Salmonella typhimurium *LT2	Styp	Styp_1
*Schizosaccharomyces pombe*	Spom	
*Shewanella oneidensis *MR-1	Sone	
*Shewanella putrifacians*	Spu	
*Staphylococcus aureus *subsp. Aureus MW2	Saur	Saur_2
*Streptococcus gordonii *str. Challis	Sgor	
*Streptococcus pneumoniae *R6	Spne	
*Streptomyces avermitilis *MA-4680	Save	
*Streptomyces caeruleus*	Scae	Scae_2
*Streptomyces coelicolor *A3(2)	Scoe	Scoe_1
*Streptomyces lavendulae*	Slav	
*Streptomyces pristinaespiralis*	Spri	
*Streptomyces roseochromogenes *subsp. Oscitans	Sros	Sros_1
*Streptomyces toyocaensis *strain 7	Stoy	
*Sulfolobus solfataricus *P2	Ssol	
*Sulfolobus tokodaii *strain 7	Stok	
*Synechococcus *sp. WH8102	SYNE_1	SYNE_1
*Synechococcus *sp. PCC7002	SYNE_2	
*Synechocystis *sp. PCC6803	SYNE_3	SYNE_3
*Thermobifida fusca*	Tfus	
*Thermosynechococcus elongates *BP-1	Telo	
*Trichodesmium erythraeum *IMS101	Tery	
*Tropheryma whipplei *TW08/27	Twhi	
*Vibrio cholerae *O1 biovar eltor strain N16961	Vcho	
*Vibrio parahaemolyticus *RIMD 2210633	Vpar	
*Wolinella succinogenes *DSM 1740	Wsu	
*Xanthomonas campestris *pv. *campestris *strain ATCC 33913	Xcam	
*Xylella fastidiosa *9a5c	Xfas	
*Yersinia enterocolitica *(type 0:8)	Yent	
*Zymomonas mobilis *subsp. *mobilis *ZM4	Zmob	

^a^The system of acronym usage is: the first letter (capital) is the first letter of the genus followed by the first three letters (lower-case) of the species. If there is no species designation the first four letters of the genu*s *are used (all in capitals). Redundant 4-letter acronyms are distinguished by unique following numbers. See [74] for a comprehensive listing with hyperlinks to the Taxonomy database records and the GenBank records at NCBI.

**Table 3:** Curated TyrA amino-acid sequence files at AroPath [35]

Complete TyrA sequences
Catalytic-core domains^a^
Pyridine-nucleotide discriminator segments^b^
NAD^+^-specific
NADP^+^-specific
Broad specificity
Cyclohexadienyl-substrate core segments
Arogenate-specific (TyrA_a_)
Prephenate-specific (TyrA_p_)
Broad specificity

TyrA_c_
TyrA_c_Δ_
Pseudogene TyrA sequences

^a^Trimmed free of N-terminal or C-terminal extensions, including any fusions with regulatory domains or other catalytic domains.

^b^High-glycine βαβ Rossmann fold at the N-terminus.

**Table 4:** Cyclohexadienyl substrates and inhibitors of TyrA proteins possess identical sidechains

**Organism**	**Co-substrate**	**Substrate(s)**	**Inhibitor(s)**^a^	**Reference**
*Synechocystis *sp.	NADP^+^	AGN	TYR	[25]
*Arabidopsis thaliana*	NADP^+^	AGN	TYR	[27, 28]
*Nitrosomonas europaea*	NADP^+^	AGN	None	[21]
*Corynebacterium glutamicum*	NAD(P)^+^	AGN	None	[42, 43]
*Neisseria gonorrhoeae*	NAD^+^	PPA^b^	HPP	[21]
*Pseudomonas stutzeri*	NAD^+^	PPA/AGN	HPP/TYR	[1]
*Pseudomonas aeruginosa*	NAD^+^	PPA/AGN	HPP/TYR	[17]
*Zymomonas mobilis*	NAD^+^	PPA/AGN	None	[18]

^a^Abbreviation: HPP, 4-hydroxyphenylpyruvate. ^b^This TyrA_c _enzyme has an overwhelming preference for PPA, but will use AGN poorly.

**Table 5:** Key to evolutionary events asserted in Figure 6

**Group**		**Evolutionary event(s) proposed**
Beta	1	Dispersal of *aroQ•pheA *> *hisH*_*b *_> *tyrA *away from one another and away from *gyrA *> *serC *and from *cmk *> *rspA *> *himD*; inversion of *aroF *with respect to *cmk*.
	2	Complete dispersal of all nine genes originally in the *gyrA*/*himD *linkage group.
	3	Insertion of *serA *after *serC*^*a*^; separation of *tyrA *and *aroF *to yield the separated 6-gene unit and 4-gene unit shown.
	4	Expulsion of *hisH*_b_from the genome; insertion of 'ORF' after *serC*.
	5	Fusion of *tyrA *with *aroF*.
	6	Loss of *hisH*_b _from genome.
Upper-Gamma	1	Insertion of *serA *after *serC*^*a*^; insertion of *aroA*_Iα _after *hisH*_b_.
	2	Translocation of *hisH*_*b *_and *tyrA *to other regions, leaving two separated 3-gene units.
	3	Fusion of *tyrA *with *aroF*.
	4	Loss of *hisH*_b_.
	5	N-terminal deletion of •*aroF *domain, and acquisition of new *aroF *gene (probable LGT).
	6	Separation of *cmk *> *rpsA *> *himD *from *aroQ•pheA *> *tyrA•aroF*.
	7	Insertion of 4 unknown genes between *gyrA *and *serC *in opposite orientation and separation of *gyrA *> ORF > ORF > ORF > *serC *from *aroQ•pheA *> *tyrA•aroF*.
	8	Loss of *himD*; translocation of *serC *away from *gyrA *and *aroQ•pheA*.

^a^Since both *Nitrosomonas *(beta Proteobacteria) and *Acidothiobacillus *(upper-gamma Proteobacteria) emerge at deep positions in the tree of Fig. 5, an almost equally parsimonius possibility is that the ancestral *serA *was retained in this syntenic position in these two genera, but was transposed elsewhere shortly after early divergence.

**Table 6:** Key to evolutionary events asserted in Figure 7

**Number**	**Evolutionary events proposed**
1	Escape of *aroQ•pheA *and *tyrA *from the ancestral *gyrA *> *serC *> *aroQ•pheA *> *hisH*_*b*_> *tyrA *> *aroF *> *cmk *> *rpsA *> *himD *supraoperon. Origin of an *aroQ•tyrA *fusion. Origin of the *aroA*_Iα_Y _> *aroQ•tyrA *operon. Addition of *tyrR*. Addition of third *aroA*_Iα _species: *aroA*_Iα_F_.
2	Fusion of *aroQ•pheA *with *aroA*_Iβ _pseudogene of unknown origin. Replacement of *hisH*_b _by *aspC *duplicate linked with three ORFs.
3	Dissociation of *gyrA *and *serC*.
4	Removal of all genes intervening between *aroQ•pheA *and *aroQ•tyrA*.
5	Dissociation of *aroF *from both *serC *and *cmk *> *rpsA *> *himD*. Insertion of *trpR *within the intervening region between *aroQ•pheA *and *aroQ•tyrA*.
6	Dissociation of *serC *> *hisH*_b _> *aroF *from *cmk *> *rpsA *> *himD*.
7	Loss of *aroA*_Iα_Y _from *tyr *operon.
8	*aroF *becomes dissociated from *hisH*_b_, and *aroA*_Iα_Y _is removed from the *tyrA *operon.
9	*ORF *> *gyrA *is inserted after *aroF*.
10	*aroQ•tyrA *becomes a pseudogene.
11	*hisH*_b _is lost.
12	*himD *is lost.
13	*cmk*, *himD *and *aroA*_Iα_Y _> *aroQ•tyrA *are lost.
14	*aroF, himD*, *aroQ•pheA*, and *aroA*_Iα_Y _> *aroQ•tyrA *are lost.
15	All intervening genes between *aroQ•pheA *and *aroQ•tyrA *are eliminated.
16	*pheA *domain of *aroQ•pheA *becomes a pseudogene.
17	Insertion of *ycaL *between *aroF *and *cmk*.
18	Insertion of ORF between *aroF *and *ycaL*.
19	Insertion of ORF between *aroQ•pheA *and *aroQ•tyrA*.

**Table 7:** Putative attenuators^a ^associated with *tyrA*

**Organism**	**Gene organization**^b^	**Fig**^c^
*Agrobacterium tumefaciens*	^¬^*hisH*_*b *_> ***tyrA***	4^d^
*Bacillus anthracis*	^¬^*aroG *> *hisH*_*b *_> ***tyrA ***> *aroF*	(11)
*Bacillus cereus*	^¬^*aroG *> *hisH*_*b *_> ***tyrA ***> *aroF*	(11)
*Bacillus halodurans*	^¬^***tyrA ***> *aroF*	(11)
*Bacteroides thetaiotaomicron*	^¬^*pheA *> *hisH*_*b *_> *aroA*_*Iβ*_•aroQ > ***tyrA***	4
*Bordetella parapertussis*	^¬^*gyrA *> *serC *> *aroQ•pheA *> ***tyrA ***> *aroF *> *cm k*> *rpsA *> *himD*	5
*Desulfovibrio vulgaris*	^¬^*aroA*' > *aroB*' > *aroQ•pheA *> *aroF *> ***tyrA ***> [*trp *operon]	4
*Enterococcus faecalis*	^¬^*aroD *> *aroA*_*Iβ *_> *aroB *> *aroG *> ***tyrA ***> *aroF *> *aroE *> *pheA*	4
*Lactococcus lactis*	^¬^*ysaA *> *blrG *> *kinG *> ***tyrA ***> *aroF *> *aroE *> *pheA*	4
*Lactobacillus plantarum*	^¬^ORF > *aroG *> *ORF *> *aroF *> ***tyrA ***> *aroE*	
*Listeria innocua*	^¬^*aroG > aroB > aroH > hisH *_*b *_> ***tyrA ***> *aroF*	4,(11)
*Streptococcus pneumoniae*	^¬^*ORF>aroC*_*I*_*>aroD>aroB>aroG>****tyrA ***> ^¬^*ORF>aroF>aroE>pheA*	4
*Thermoanaerobacter tencongensis*	^¬^*pheA > aroA*_*I*β _> ***tyrA ***> *aroF *> *ORF *> *ORF*	
*Thermus thermophilus*	^¬^*aroA*_*Iβ *_> ***tyrA***	
*Vibrio parahaemolyticus*	^¬^*aroA*_*Iα_Y *_> ***tyrA***	6
*Vibrio vulnificus*	^¬^*aroA*_Iα_Y _> ***tyrA***	6

^a^Attenuators were extracted from the website of Merino [66]. Links are provided for viewing the complete data, including a visualization of the putative attenuator structures.

^b^The symbol ^¬ ^is used for attenuators. Genes encoding the alternative biochemical steps that were recently reported for formation of dehydroquinate from aspartate semialdehyde and ketohexose 1-phosphate [52] are designated *aroA*' and *aroB*'.

^c^Refers to figures within this manuscript or, if enclosed within parentheses, to the figure in ref. [7].

^d^See the consensus gene organization for α Proteobacteria.
