# Supplementary material for: The TyrA family of aromatic-pathway dehydrogenases in phylogenetic context
Source: BMC Biol. 2005 May 12;3:13. doi: 10.1186/1741-7007-3-13 (PMC1173090; doi:10.1186/1741-7007-3-13)
Supplement: Additional File 1 — Table S1, entitled "Key to organism acronyms and sequence identifiers", is provided as supplementary material in an html document. This table contains the full collection of sequence data and annotations contained in this paper, and gene identification (gi) numbers are included and hyperlinked to facilitate access to the corresponding GenBank records. For future reference to a progressively updated table, refer to the AroPath website [73]. [file 1741-7007-3-13-S1.html]

TableS1--Key to organism acronyms and sequence identifiers


| Table S1. Key to organism acronyms and sequence identifiers | | | |
| --- | --- | --- | --- |
| --- | | | |
| Abbreviation\* | Organism | TyrA     *gi* number | TyrR     *gi* number |
| --- | | | |
| Aact | *Actinobacillus actinomycetemcomitans* | NA | NA |
| ACIN\* | *Acinetobacter*  sp. | 50085330 | NA |
| ANAB\* | *Anabaena* sp. | 17228636 | NA |
| ANAB\* | *Anabaena* sp. | 17227914 | NA |
| Atha\* | *Arabidopsis thaliana* | 16903100 | NA |
| Aful\_1\* | *Archaeoglobus fulgidus* | 11497843 | NA |
| Atum\* | *Agrobacterium tumefaciens* | 17937318 | NA |
| Avin | *Azotobacter vinelandii* | 53612601 | NA |
| Bant\* | *Bacillis anthracis* | 21400838 | NA |
| Bcer\* | *Bacillus cereus* | 30021055 | NA |
| Bhal\_2\* | *Bacillus halodurans* | 15614229 | NA |
| Bste | *Bacillus stearothermophilus* | NA | NA |
| Bsub\* | *Bacillis subtilis* | 16079318 | NA |
| Bthu\* | *Bacillus thuringiensis* israelensis | 49479572 | NA |
| Blon\_1\* | *Bifidobacterium longum* | 23465944 | NA |
| Bflo\* | *Blochmannia floridanus* | 33519649 | NA |
| Bbro | *Bordetella bronchisepticus* | NA | NA |
| Bcep\* | *Burkholderia cepacia* | NA | NA |
| Bfun\_1 | *Burkholderia fungorum* | 48784756 | NA |
| Bmal\* | *Burkholderia mallei* | 53724789 | NA |
| Bpse\_6\* | *Burkholderia pseudomallei* | 53720127 | NA |
| Cjej\* | *Campylobacter jejuni* | 15791518 | NA |
| Cvio\* | *Chromobacterium violaceum* | 34104714 | NA |
| Ceff\* | *Corynebacterium efficiens* | 25026751 | NA |
| Cglu\_1\* | *Corynebacterium glutamicum* | 19551477 | NA |
| Ddes | *Desulfovibrio desulfuricans* | 53692015 | NA |
| Dvul\* | *Desulfovibrio vulgaris* |  |  |
| Dace\_5 | *Desulfuromonas acetoxidans* | NA | NA |
| Efae\_1\* | *Enterococcus faecalis* | 12407966 | NA |
| Efae | *Enterococcus faecium* | 22991440 | NA |
| Ecar\* | *Erwinia carotovora* | 50122273 | 50120916 |
| Ecol\_1\* | *Escherichia coli* | 26248963 | 25301105 |
| Gmet | *Geobacter metallireducens* | 48846797 | NA |
| Gsul\* | *Geobacter sulfurreducens* | 39997701 | NA |
| Gvio\* | *Gloeobacter violaceus* | 37519975 | NA |
| Hinf\* | *Haemophilus influenzae* | 16273204 | 16272359 |
| Hhep\* | *Helicobacter hepaticus* | 32262543 | NA |
| Hpyl\* | *Helicobacter pylori* | 15645990 | NA |
| Kpne | *Klebsiella pneumoniae* | NA | NA |
| Lesc | *Lycopersicon esculentum* | NA | NA |
| Linn\* | *Listeria innocua* | 16801104 | NA |
| Lmon\* | *Listeria monocytogenes* | 16803963 | NA |
| Lcor\_3 | *Lotus corniculatus var. japonicus* | NA | NA |
| Mthe\_7\* | *Methanothermobacter thermoautotrophicus* | 15679635 | NA |
| Mjan | *Methanococcus jannaschii* | 15668793 | NA |
| Mkan\_1\* | *Methanopyrus kandleri* AV19 | 20094235 | NA |
| Mbar | *Methanosarcina barkeri* | 23051388 | NA |
| Mdeg | *Microbulbifer degradans* | 23026646 | NA |
| Mtub\* | *Mycobacterium tuberculosis* | 15843374 | NA |
| Mxan | *Myxococcus xanthus* | NA | NA |
| Ngon | *Neisseria gonorrhoeae* | NA | NA |
| Neur\* | *Nitrosomonas europaea* | 22954363 | NA |
| Npun\_1 | *Nostoc punctiforme* | 23124347 | NA |
| Npun\_1 | *Nostoc punctiforme* | 23128268 | NA |
| Naro | *Novosphingomonas aromaticivorans* | 23108542 | NA |
| Oihe\* | *Oceanobacillus iheyensis* HTE831 | 23099236 | NA |
| Osat\* | *Oryza sativa* ssp. japonica | NA | NA |
| Pagg | *Pantoea agglomerans* | 267186 | 7388342 |
| Pmul\* | *Pasteurella multocida* | 15602529 | 12721224 |
| Plum\* | *Photorhabdus luminescens* | 37525230 | 37526471 |
| Plum\* | *Photorhabdus luminescens* | 37527434 | NA |
| Pmar\_3\* | *Prochlorococcus marinus* CCMP1378 MED4 | 33862121 | NA |
| Pmar\_10\* | *Prochlorococcus marinus* MIT9313 | 33863991 | NA |
| Paer\_1\* | *Pseudomonas aeruginosa* | 15598360 | NA |
| Pflu | *Pseudomonas fluorescens* | 23061127 | NA |
| Pput\* | *Pseudomonas putida* | 26988501 | NA |
| Pstu | *Pseudomonas stutzeri* | 5712093 | NA |
| Reut | *Ralstonia eutropha* | NA | NA |
| Rsol\* | *Ralstonia solanacearum* | 17545625 | NA |
| Rcap | *Rhodobacter capsulatus* | NA | NA |
| Rsph | *Rhodobacter sphaeroides* | 22957104 | NA |
| Rpal\* | *Rhodopseudomonas palustris* | 22961616 | NA |
| Rrub\_1 | *Rhodospirillum rubrum* | 22967332 | NA |
| Scer\* | *Saccharomyces cerevisiae* | 6319643 | NA |
| Styp\_1\* | *Salmonella typhimurium* | 16765984 | 16420210 |
| Spom\* | *Schizosaccharomyces pombe* | 19076029 | NA |
| Sput | *Shewanella putrefaciens* | NA | NA |
| Saur\_2\* | *Staphylococcus aureus* | 21282981 | NA |
| Sgor | *Streptococcus gordonii* | NA | NA |
| Spne\* | *Streptococcus pneumoniae* | 15903274 | NA |
| Scoe\_1\* | *Streptomyces coelicolor* | 24413756 | NA |
| Scoe\_1\* | *Streptomyces coelicolor* | 7481118 | NA |
| Spri | *Streptomyces pristinaespiralis* | 1575337 | NA |
| Stoy\* | *Streptomyces toyocaensis* | 21911427 | NA |
| Ssol\* | *Sulfolobus solfataricus* | 15897245 | NA |
| SYNE\_1\* | *Synechococcus* sp. WH8102 | 33866590 | NA |
| SYNE | *Synechococcus* sp. PCC7002 | NA | NA |
| SYNE\_3\* | *Synechocystis* sp. PCC6803 | 16330562 | NA |
| Telo\* | *Thermosynechococcus elongatus* | 22299978 | NA |
| Tfer\_1 | *Thiobacillus ferrooxidans* | NA | NA |
| Tery | *Trichodesmium erythraeum* | 48892373 | NA |
| Vcho\* | *Vibrio cholerae* | 15640715 | 15641320 |
| Vpar\_2\* | *Vibrio parahaemolyticus* | 28897321 | 28898643 |
| Wsuc\* | *Wolinella succinogenes* | 34556783 | NA |
| Xcam\* | *Xanthomonas campestris* | 21230932 | NA |
| Xfas\* | *Xylella fastidiosa* (almond strain) | 28199252 | NA |
| Yent | *Yersinia enterocolitica* | NA | NA |
| Ypes\* | *Yersinia pestis* KIM | 22124813 | NA |
| Zmob\* | *Zymomonas mobilis* | 56551316 | NA |
| --- | | | |
| **\*** Complete genomes are indicated by asterisks. | | | |
